# Effects of global change on snakebite envenoming incidence up to 2050: a modelling assessment

**DOI:** 10.1016/S2542-5196(24)00141-4

**Published:** 2024-08-07

**Authors:** Gerardo Martín, Joseph James Erinjery, Dileepa Ediriweera, Eyal Goldstein, Ruchira Somaweera, H Janaka de Silva, David G Lalloo, Takuya Iwamura, Kris A Murray

**Affiliations:** a Departamento de Sistemas y Procesos Naturales, Escuela Nacional de Estudios Superiores Unidad Mérida, Universidad Nacional Autónoma de México, Mérida, Mexico; b MRC Centre for Global Infectious Disease Analysis, Department of Infectious Disease Epidemiology, School of Public Health, Imperial College London, London, UK; c School of Zoology, Department of Life Sciences, Tel Aviv University, Tel Aviv, Israel; d Department of Zoology, Kannur University, Kannur, India; e Department of Medicine, Faculty of Medicine, University of Kelaniya, Ragama, Sri Lanka; f Centre for Health Informatics, Computing, and Statistics, Lancaster University Medical School, Lancaster, UK; g Ecosystem Modelling, University of Göttingen, Göttingen, Germany; h Aquatic and Subterranean Ecology, Stantec Australia, Perth, WA, Australia; i Department of Clinical Sciences, Liverpool School of Tropical Medicine, Liverpool, UK; j Institute for Environmental Sciences, University of Geneva, Geneva, Switzerland; k MRC Unit The Gambia at the London School of Hygiene and Tropical Medicine, Fajara, The Gambia; l Centre on Climate Change and Planetary Health, London School of Hygiene and Tropical Medicine, London, UK

## Abstract

**Background:**

Human activities are driving climate, land cover, and population change (global change), and shifting the baseline geographical distribution of snakebite. The interacting effects of global change on snakes and communities at risk of snakebite are poorly understood, limiting capacity to anticipate and manage future changes in snakebite risk.

**Methods:**

In this modelling study, we projected how global change will affect snakebite envenoming incidence in Sri Lanka, as a model system that has a high incidence of snakebite. We used the shared socioeconomic pathway (SSP) scenario analysis framework to integrate forecasts across the domains of: climate change (historical trend from WorldClim plus three underlying regional circulation models [RCMs] in the Coordinated Regional Downscaling Experiment-South Asia repository, with two emissions pathways [representative concentration pathways RCP4.5 and RCP8.5]); land cover change (Dyna-CLUE model); and human population density change (based on Gridded Population of the World data) from Jan 1, 2010 to Dec 31, 2050. Forecasts were integrated under three different development scenarios: a sustainability pathway (SSP1 and no further emissions), a middle-of-the-road pathway (SSP2 and RCP4.5), and a fossil-fuelled pathway (SSP5 and RCP8.5). For SSP2 and SSP5, we nested three different RCMs (CNRM-CM5, GFDL-CCM3, and MPI-ESM-LR; mean averaged to represent consensus) to account for variability in climate predictions. Data were used as inputs to a mechanistic model that predicted snakebite envenoming incidence based on human–snake contact patterns.

**Findings:**

From 2010 to 2050, at the national level, envenoming incidence in Sri Lanka was projected to decrease by 12·0–23·0%, depending on the scenario. The rate of decrease in envenoming incidence was higher in SSP5-RCP8.5 than in SSP1 and SSP2-RCP4.5. Change in envenoming incidence was heterogenous across the country. In SSP1, incidence decreased in urban areas expected to have population growth, and with land cover changes towards anthropised classes. In SSP2-RCP4.5 and SSP5-RCP8.5, most areas were projected to have decreases in incidence (SSP5-RCP8.5 showing the largest area with incidence reductions), while areas such as the central highlands and the north of the country showed localised increases. In the model, decreases occurred with human population growth, land use change towards anthropised classes (potentially shifting occupational risk factors), and decreasing abundance of some snake species, potentially due to global warming and reduced climatic and habitat suitability, with displacement of some snake species.

**Interpretation:**

Snakebite envenoming incidence was projected to decrease overall in the coming decades in Sri Lanka, but with an apparent emerging conflict with sustainability objectives. Therefore, efforts to mitigate snakebite envenoming incidence will need to consider the potential impacts of sustainability interventions, particularly related to climate and land use change and in areas where increases in incidence are projected. In view of global change, neglected tropical diseases and public health issues related to biodiversity, such as snakebite, should be managed collaboratively by both environment and health stakeholders.

**Funding:**

UK Medical Research Council.

## Introduction

Snakebite envenoming is a neglected tropical disease, the incidence of which is associated with the natural environment and particular social conditions, making it difficult to control.^1^ Climate is known to influence snakebite risk,^2^, ^3^ although it is one among many potential causal factors undergoing rapid change. Climate, land use, and human population changes (hereafter referred to as global change) are defining a new era in Earth's history caused by human activity.^4^ Global change has been facilitating the geographical expansion of some neglected tropical diseases,^5^, ^6^ possibly including snakebite,^1^, ^3^ while likely restricting others.^7^ To mitigate the health hazards associated with these changes, we need to better understand how global change could interactively affect snakebite burden.

**Research in context**

**Evidence before this study**

Snakebite is frequently associated with climate, land cover, and social factors (eg, poverty and occupation, and population density), which has generated wide interest around how snakebite risk is being affected by climate, land cover, and population change (which we refer to as global change). We searched Google Scholar for articles published in English before Dec 31, 2019, using the search terms “snakebite risk”, “snakebite and climate change”, “snakebite and land cover change”, “spatial modeling of snakebite risk”, “occupation and land cover”. Many previous studies have projected that climate change is likely to increase snakebite risk by altering snake distributions or activity patterns; however, few have simultaneously assessed change or interactions with other factors such as social and land cover features.

**Added value of this study**

We used a mechanistic model of snakebite envenoming incidence in Sri Lanka to explore how climate, land cover, and human population growth will together affect snakebite envenoming incidence up to 2050 according to various contrasting scenarios of social and environmental change. To the best of our knowledge, this study is the first to incorporate three central components of global change into a single mechanistic framework to predict snakebite envenoming incidence. We show that by the year 2050 in Sri Lanka, snakebite envenoming incidence could decrease in tropical lowlands and increase in the highlands due to changes in the distribution and abundance of venomous snake species, which affect future human–snake contact and envenoming patterns. Countrywide, incidence was projected to decrease the most in the least sustainable scenarios, highlighting an apparent conflict between the objectives of human health and sustainable development.

**Implications of all the available evidence**

The mechanisms underlying snakebite envenoming incidence and the likely range of climate change, land cover, and human population conditions suggest that snakebite envenoming incidence will increase locally (particularly in the highlands) but decrease considerably at a national scale in Sri Lanka by 2050. This result is likely to be fundamentally driven by the key factors underpinning loss of biodiversity, the mitigation of which is independently the focus of local, national, and global sustainability and conservation initiatives. Joint efforts between public health and conservation agencies are therefore needed to protect both human health and biodiversity. Potential solutions include increasing epidemiological surveillance, prevention through educational campaigns for specific socioecological contexts to improve the uptake of and access to protective equipment, and access to effective therapeutics.

Most existing attempts to predict future snakebite incidence consist of analyses of future snake geographical distributions, and broadly indicate that climatic conditions will improve for some key species, particularly at high latitudes and elevations as the climate warms.^3^ In Sri Lanka, our study system, previous predictions have been based on analyses associating snakebite incidence statistics with climatic predictors, and suggest that by the year 2050, incidence could increase by around 31% due to changes in rainfall (hypothesised to primarily affect snake activity patterns).^2^ These approaches have provided important insights, but attempts to integrate across climate conditions, land use, and population growth to project future snakebite risk remain absent.

The mechanisms by which climate, land cover, and population affect snakes and snakebite are unclear. Climate affects snake physiology, as snakes are ectotherms.^8^ Land cover relates directly to snake habitat preferences,^9^ and human population density is the principal cause of land use change and is also the population at risk.^10^ In addition, land cover and its use is a surrogate of occupation and therefore occupational risks.^11^, ^12^ Climate also affects habitat types, human physiology, and agricultural practices that relate directly to snakebite risk.^13^ Evidence from analyses of human–vertebrate interactions suggests that epidemiological changes in disease risks are likely to be diversely affected by many dimensions of global change simultaneously.^14^

Among climate-sensitive diseases, environmental and social changes are affecting burdens, transmission dynamics, and geographical distributions.^5^, ^15^ For example, increasing temperatures and forest loss are facilitating the movement of malaria vectors further north and south of the tropics and to high elevations, increasing malaria burden in locations previously unsuitable, while decreasing it in others.^16^ For neglected tropical diseases such as dengue and Chagas, poverty and growing inequality are partially responsible for the increased burden in recent decades, but climate change is also likely to be playing a role.^15^ In addition to environmental change, economic inequality and poverty are also well known risk factors for snakebite,^12^ further highlighting the need for comprehensive predictive frameworks under the remit of global change.

For Sri Lanka, analyses in the past decade have produced high-quality data on venomous snake abundances for multiple species with use of point process models, and on snakebite envenoming incidence with use of model-based geostatistics.^17^, ^18^ Both products were the basis of a mechanistic model for snakebite that incorporated the three main axes of global change: climate, land use change, and population growth.^19^ However, high-resolution spatiotemporal snakebite forecasts under scenarios of global change are absent worldwide. In the present study we addressed this key gap. We integrated information across data domains to project snakebite envenoming incidence in Sri Lanka up to 2050, under contrasting development trajectories, following a routinely employed scenario analysis approach based on the shared socioeconomic pathways (SSPs).^20^ We explored three contrasting development scenarios: a sustainability pathway (SSP1 with no further greenhouse gas emissions), a middle-of-the-road pathway (SSP2 with moderate emissions reductions), and a fossil-fuelled pathway (SSP5 with limited emissions reductions).

## Methods

### Model overview

**Figure 1:**
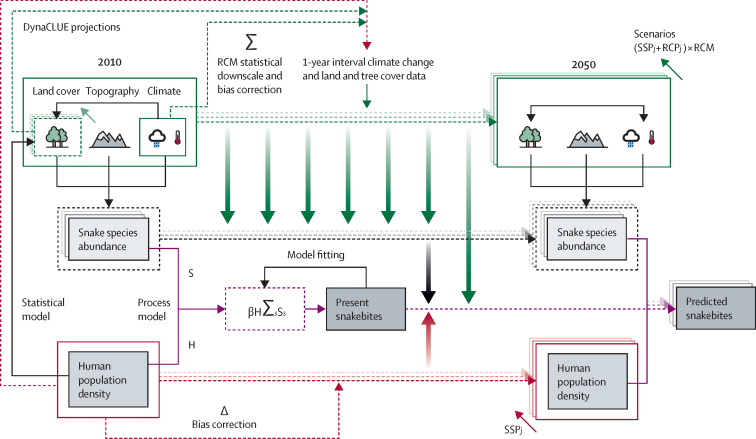
Diagram of the workflow to develop the data-based and process-based model for snakebite envenoming RCM=regional circulation model. SSP=shared socioeconomic pathway. RCP=representative concentration pathway. *j*=individual SSP (SSP1, SSP2, or SSP5). S=snake abundance. H=human population density. β=human–snake contact rate. *s*=individual snake species.

Envenoming incidence predictions were based on combining process-based and statistical models, transferred to a range of global change scenarios.^19^ A model overview is provided in figure 1. The snakebite envenoming model was used to estimate snakebite envenoming incidence at 5 km^2^ resolution in Sri Lanka, annually from Jan 1, 2010 to Dec 31, 2050, with use of three main data sources: (1) abundance patterns of the seven medically relevant (venomous) land snakes (*Bungarus caeruleus*, *Bungarus ceylonicus*, *Daboia russelii*, *Echis carinatus*, *Hypnale spp* [three species complex: *H hypnale*, *H nepa*, and *H zara*], *Naja naja*, and *Trimeresurus trigonocephalus*), which were independently estimated with point process models at 1 km^2^ resolution;^18^ (2) human population density, with use of published estimates of the spatial variability of population density^21^ at 1 km^2^ resolution; and (3) land cover information with five distinct land cover classes (forest, degraded forest, agriculture, urban, and tea plantation), which were predicted annually with use of the Dyna-CLUE model^22^ at 1 km^2^ resolution. In Dyna-CLUE, climate and human population density were also predictors of land cover change. Several land cover-derived variables (eg, proportion of agriculture, distance to forests, and percentage of tree cover) were inputs into the snake abundance models.^18^ Details of each step are described herein.

### Global change scenarios

The uncertainty in future trajectories of climate change and socioeconomic development, and their consequent impacts on the population, the environment, and ultimately snakebite incidence, were represented with a routinely used SSP scenario modelling framework. We evaluated different SSPs (SSP1, SSP2, and SSP5) combined with different emissions pathways (no further emissions, representative concentration pathway [RCP] 4.5, and RCP8.5), representing three different scenarios from more to less sustainable (sustainability scenario [SSP1 and no further emissions], middle-of-the-road scenario [SSP2 and RCP4.5], and fossil-fuelled development scenario [SSP5 and RCP8.5]; figure 1). The climate component of the models came from either the historical trend (from WorldClim data, version 2.0) with a simple future projection to represent no further emissions, or one of three downscaled regional circulation models (RCMs), representing a range of radiative forcings of anthropogenic emissions and taken from the Coordinated Regional Downscaling Experiment-South Asia (CORDEX-SA) climate data repository. Therefore, for the three main scenarios we used seven distinct projections in total (sustainability: one projection, comprising SSP1 plus historical climate; middle-of-the-road: projections with three climate models comprising each of three RCMs plus SSP2-RCP4.5; and fossil-fuelled development: projecitons with three climate models comprising each of three RCMs plus SSP5-RCP8.5).

### Snakebite incidence and snake abundance

The snakebite incidence model comprises two parts, the bite and the envenoming. Bites are represented as a mass-action process, in which snakes and humans mix homogeneously in each spatial unit, with a contact rate dependent on land cover class as a surrogate of human risk factors, snake species, and a conditional autoregressive function of space:

$$\begin{array}{c} \Delta H_{e}=H_{s,t}\times \left(1-\exp\left(-\beta \left(H_{total},L\right)\sum_{S=1}^{n}c_{s}S_{s,t}+\rho_{i,-i}\right)\right)\times P_{env}\\ \log\left(\frac{P_{env}}{1-P_{env}}\right)=B_{L}+\sum_{s=1}^{n}b_{s}S_{s} \end{array}$$

where *ΔH*_e_ is total snakebite envenoming incidence (new envenoming cases per unit time) and *H*_s,t_ is the susceptible human population at time *t*. β(*H*_total_*,L*) is the human–snake contact rate, which is a function of land cover and 2010 human population density (appendix p 11). The result is a contact rate that varies in space and partly corrects snake abundance in relation to human population density.^19^ *c*_s_ is a species-specific contact rate that summarises factors such as aggressiveness and activity overlap with humans and how these affect envenoming incidence, *S*_s_ is the potential abundance of each snake species *s* (n=7; 17 parameters), and *P*_env_ is the probability that a bite results in envenoming (function of land cover, *B*_L_, and snake species, *b*_s_, 13 parameters in total). Finally, ρ_i,–i_ is the conditional autoregressive random effect. All parameters were estimated with Bayesian Markov chain Monte Carlo sampling.

This model was selected from a set of six different base models in which we gradually added land cover, human population density, and independent expert information on snake species biology. The selected model minimised the deviance information criterion, improved posterior predictive checks for snakebite envenoming incidence (Pearson's correlation test corrected for spatial correlation: *r*=0·85, Monte Carlo p=0), and had residuals with a mean value of zero. Full details of the final model have been published previously.^19^

The variables representing snake populations are approximations of abundance patterns estimated with area-interaction point process models, thus do not actually represent population numbers but rather an index of abundance. The estimated indices were adjusted for each snake species’ relative abundance by independent expert input. Point process models include part of the effect of climate and land cover on the snakebite process, as they are functions of climate, and several land cover-derived variables (eg, proportion of agriculture, distance to forests, and percentage of tree cover) and spatial effects. To account for ecological plasticity of each species, we used available species occurrence data from the Global Biodiversity Information Facility from outside Sri Lanka (data extracted Feb 21, 2019), from published data for Sri Lanka,^23^ and from private herpetologist collections from Sri Lanka (via author RS) to estimate ecological niches, represented as distance from each pixel's climatic conditions to the species-wide centroid. The centroid was the mean of the climatic conditions where snakes have been observed to be present. Full details of the approximation of abundance have been published previously.^18^

### Global change data

All model projection data were downscaled specifically for this study. Downscaling requires bias corrections by comparing conditions generated by the forecasting methods with observed environmental conditions. We used two methods to obtain bias correction factors with the modified delta method:^24^ difference and ratio. Difference factors consisted of subtracting predicted from observed (used for temperature), and the ratio was observed to predicted (used for rainfall, tree cover, and population). These correction factors were applied to future conditions to interpolate differences and then convert changes back to variable values (appendix p 1).

For the SSP1 scenario, we assumed there would be no or minimal further emissions to drive future climate change. Since no RCP2.6 scenario (which represents ambitious emissions reductions) was available in CORDEX-SA, we instead used WorldClim data based on the trends observed between 1970 and 2010. For SSP2, assumed future greenhouse gas concentrations correspond to RCP4.5, and SSP5 corresponds to RCP8.5 (appendix p 4).^24^

Predictions from different RCMs are variable due to the approaches to force the effect of anthropogenic emissions. To account for this variation, we selected the three most reliable Coupled Model Intercomparison Project Phase 5 (CMIP5) RCMs according to previous assessments.^25^ We downscaled three CMIP5 CORDEX-SA RCMs (National Centre for Meteorological Research Coupled Model 5 [CNRM-CM5], Geophysical Fluids Dynamics Laboratory Coupled Model 3 [GFDL-CM3], and Max Plank Institute Earth System Model lower resolution [MPI-ESM-LR]) with the delta method^26^ to match the resolution of the snake point process models. The RCM-predicted variables were monthly minimum and maximum mean temperature (taken as the mean of daily maximum and minimum temperatures of each month) and total precipitation (ie, total rainfall) and downscaled from 55 × 55 km to 1 × 1 km for the years 1970–2050. These data provided the RCP4.5 and RCP8.5 future climate scenarios. To validate the downscaled data, we measured the Pearson's correlation, linear regression slope, intercept, and root mean squared error between the downscales of 1970–2006 (CMIP5 reference period) with observed climates of the Climate Research Unit (version 4.03; hosted by the University of East Anglia [Norwich, UK] and the UK National Centre for Atmospheric Science) for the same period (appendix p 1).

We developed a layer of land cover with five classes (forest, degraded forest, agriculture, urban, and tea plantation) for the year 2010 at 1 km^2^ resolution as a starting point for land cover change predictions (appendix p 11). The methods used to generate these five classes of land cover layer are provided in the appendix (p 2). Annual pixel-wise land cover changes were simulated with Dyna-CLUE (version 2.0;^22^ DC2). To simulate land cover changes from the starting layer with DC2, we developed five inputs: (1) class transition rules; (2) annual rates of change between all pairs of land cover classes; (3) location factors (probability of the presence of a specific land cover class, estimated by logistic regression of the presence or absence of each land cover class with a series of dynamic and static factors; appendix pp 5–7); (4) demand for each class; and (5) spatial restrictions. Each of the steps are fully described in the appendix (pp 2–3).

Our tree cover predictions (baseline cover retrieved from the Global Land Cover Facility for the year 2010, presented in the appendix [p 14]) were also obtained with DC2, following the same methodological steps used for land cover. We began by classifying tree cover data in five categories, representing 20% intervals of the proportion of land covered by trees: 0–20%, 21–40%, 41–60%, 61–80%, and 81–100%. Details on each of the inputs required by DC2 are fully described in the appendix (pp 2–3). To ensure compatibility of tree cover predictions with the data used in the snake distribution models,^18^ we estimated a correction factor to reclassify the categorical tree cover predictions from five categories to proportions (0·00–0·99; appendix p 3).

We obtained 1 km^2^ downscaled projected human population density data for the years 2010, 2020, 2030, 2040, and 2050 reported by Gao,^21^ based on Jones and O’Neill's^20^ data (baseline density presented in the appendix [p 11]). To make the SSP1, SSP2, and SSP5 scenarios compatible for projection with the Gridded Population of the World (GPW; version 4) data used for the mechanistic model,^19^ we estimated a bias-correction factor by calculating relative annual change, using the GPW (version 4) data from Jan 1, 2005, to Dec 31, 2010 from WorldPop as the reference and the downscaled predictions for the same years. The latest population estimates from GPW were up to Dec 31, 2015. The method used to correct bias of all population projections after 2015 was by calculating the mean quotient of observed to predicted populations (same as used for rainfall and tree cover). To obtain annual pixel-wise human population values we interpolated the bias-corrected population with piecewise Hermite polynomials for every year between the intervals 2010–2020–2030–2040–2050.^24^

### Snakebite projections

The models of snake abundance used to fit the projected model were generated with long-term climatic conditions. To identify the temporal span of annual climate relevant to snakebite incidence, we re-analysed Ediriweera and colleagues’^17^ snakebite survey incidence data with continuous bioclimatic variables (annual means of monthly minimum and maximum temperature and total rainfall) mean averaged for the year of the population survey (2013), and for the 1–10 preceding years. The climate average that best explained snakebite incidence was the average climate of 2011–13. Thus, we projected the snake abundance models using the average climate of each year and the two preceding. To feed the snakebite envenoming model with predictions of snake abundance, human population density, and land cover, we upscaled the 1 km^2^ predictions to 25 km^2^ by aggregating snake point intensity and population density; land cover predictions were upscaled by majority vote, assigning the class to the upscaled area depending on which class covered the largest area in the 1 km^2^ area.

To estimate annual percentage change in snakebite envenoming incidence we did linear regressions between incidence rates and year for the projections within each SSP-RCP scenario in each spatial unit. With the predicted incidence rates by year we calculated annual percentage change versus baseline year 2010, and determined mean change at the national scale. Annual percentage changes were accumulated up to 2050, and overall percentage change between 2010 and 2050 was calculated as (2050 envenoming incidence – 2010 envenoming incidence)/2010 envenoming incidence). To estimate the spatial trend in envenoming incidence change, we regressed the pixel-wise predicted annual envenoming incidence estimates against time to obtain the rate of envenoming incidence change, and calculated the percentage change between 2010 and 2050. To represent consensus among the nested RCMs within SSP-RCP scenarios we calculated the mean of the percentage change up to year 2050. Finally, to identify consensus among RCMs, we did a pixel-wise regression of incidence against time, and thresholded the resulting maps to identify areas with decreasing incidence (negative coefficients).

All analyses were done in R (version 4.0). For operations with raster data we used the raster package (version 2.8-19), and point process models were projected with the spatstat package (version 1.58-2). The Bayesian model was generated with the package nimble (version 0.12.2).^27^

### Role of the funding source

The funder of the study had no role in study design, data collection, data analysis, data interpretation, or writing of the report.

## Results

Validation based on backcasts showed that all the downscales complied with the minimum requirements: positive correlation with observed climate, correlation coefficient (*r*) close to 1, linear regression slope coefficient close to 1 and significantly different from 0, and intercept non-significantly different from 0. The performance of temperature predictions was higher than for rainfall predictions (appendix p 9).

**Figure 2:**
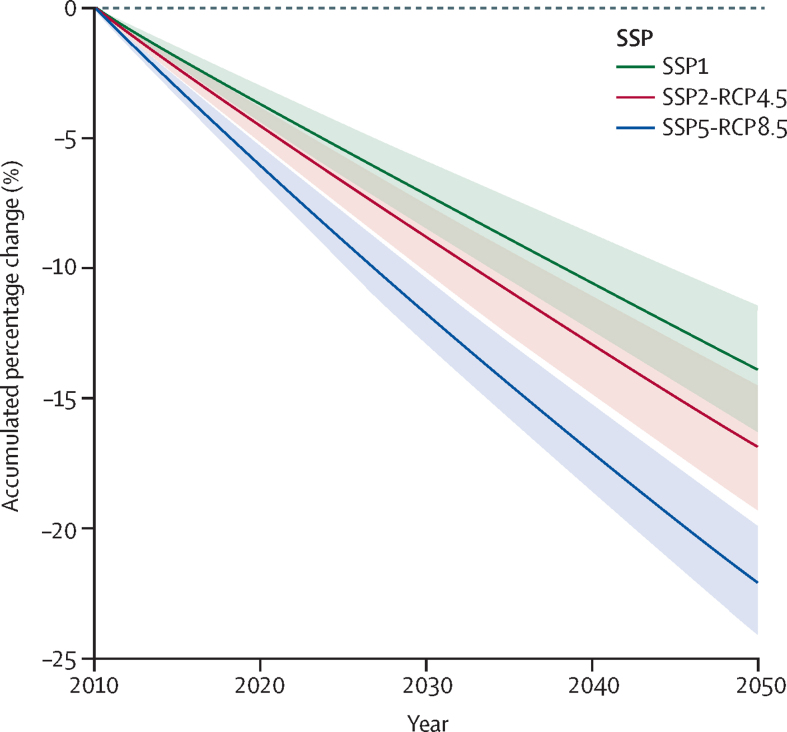
Accumulated percentage change in national mean envenoming incidence between 2010 and 2050 in each SSP scenario The shaded regions represent the standard error as obtained from mean squared error for each scenario regression coefficient. SSP=shared socioeconomic pathway. RCP=representative concentration pathway.

Our results showed that the modelled interaction between climate, land cover, and population growth overall was associated with reduced snakebite envenoming incidence over time in Sri Lanka. When mean-averaged nationally, envenoming incidence decreased in all SSP scenarios from 2010 up to 2050, suggesting that regardless of the uncertainty of future climate, land cover, and population predictions, snakebite envenoming incidence is likely to decline in Sri Lanka overall when all factors are integrated (figure 2). The rate of decrease in envenoming incidence was lower in SSP1 (sustainability pathway) and SSP2-RCP4.5 (middle-of-the-road pathway) than in SSP5-RCP8.5 (fossil-fuelled pathway; figure 2). Some variation was also evident across the three RCMs used (appendix p 12). Comparing the models, intermediate rates of decrease were observed in SSP2-RCP4.5 and SSP5-RCP8.5 under the CNRM-CM5 climate scenario. The fastest rates of decrease occurred in SSP2-RCP4.5 under the GFDL-CM3 climate scenario, and in SSP5-RCP8.5 under the GFDL-CM3 and MPI-ESM-LR climate scenarios. The rate of decrease for SSP5-RCP8.5-CNRM-CM5 was lower than for SSP2-RCP4.5-GFDL-CM3 (appendix p 12). However, under SSP2-RCP4.5 we observed high variability, largely due to the difference between the SSP2-RCP4.5 and SSP5-RCP8.5 MPI-ESM-LR scenarios (appendix pp 10, 12). Across the RCMs, the projected decrease in envenoming incidence for the 41-year period varied from 12·0% (SSP2-RCP4.5 MPI-ESM-LR) to 23·0% (SSP5-RCP8.5 GFDL-CM3; appendix p 10). Human population growth during 2010–50 was highest for SSP2-RCP4.5, followed by SSP1, and lowest for SSP5-RCP8.5 (appendix p 22).

**Figure 3:**
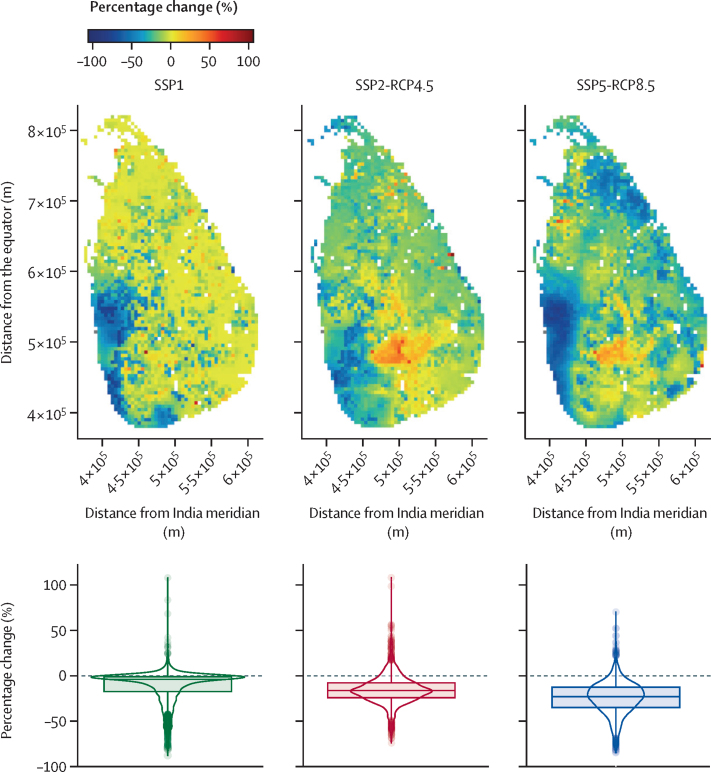
Predicted percentage change in envenoming incidence between 2010 and 2050 in each SSP scenario Maps show the predicted percentage change based on the pixel-wise annual expected incidence change, obtained as: (2050 envenoming incidence – 2010 envenoming incidence) / 2010 envenoming incidence). The box and violin plots show the countrywide distribution of predicted percentage change, where the central horizontal lines indicate the median, box limits indicate IQR, error bars indicate non-parametric 95% CIs, and circles are outliers of the 95% CIs. Each boxplot is located underneath its corresponding map. Most areas with predicted increasing incidence are located in the central highlands of Sri Lanka (appendix p 14). Geographical coordinates are from the SLD99 coordinate system for Sri Lanka; the x-axis origin is the central meridian of India. SSP=shared socioeconomic pathway. RCP=representative concentration pathway.

**Figure 4:**
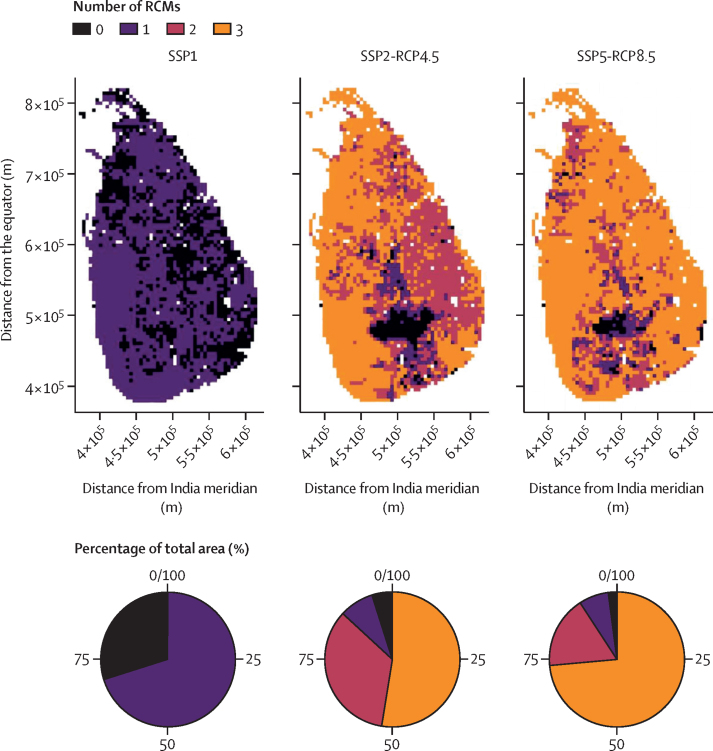
Areas where envenoming incidence is predicted to decrease by 2050 in each SSP scenario Colours indicate the number of RCMs with agreement on decreasing incidence (CNRM-CM5, GFDL-CM3, MPI-ESM-LR, or WorldClim only for SSP1). Pie charts indicate the percentage of the total area with decreased incidence by agreement between RCMs (eg, the orange section in the chart for SSP2 shows that incidence would decrease in >50% of the area under all three RCMs). Black areas show complete agreement between RCMs that incidence would be either stable or increasing (ie, no RCMs with decreasing incidence; for SSP1, the black areas indicate where incidence did not decrease under the WorldClim trend). Geographical coordinates are from the SLD99 coordinate system for Sri Lanka; the x-axis origin is the central meridian of India. SSP=shared socioeconomic pathway. RCP=representative concentration pathway. RCM=regional circulation model.

Despite the national trends, envenoming incidence and its change were heterogeneous across the country between 2010 and 2050. In SSP1, incidence remained mostly stable, with decreases in the west and southwest (overlapping with urban areas likely to have population growth; appendix pp 11, 22) and increases in small and isolated areas (figure 3). In SSP2-RCP4.5 and SSP5-RCP8.5, although most areas were projected to have decreases in incidence, in some areas incidence was projected to increase, such as in the central highlands and the north of the country, by as much as 100% (figure 3; changes by RCM are provided in the appendix [p 12]). Contrary to the national average, increasing local incidence was particularly evident in the middle-of-the-road scenario (SSP2-RCP4.5; figure 3, figure 4, appendix p 12).

**Figure 5:**
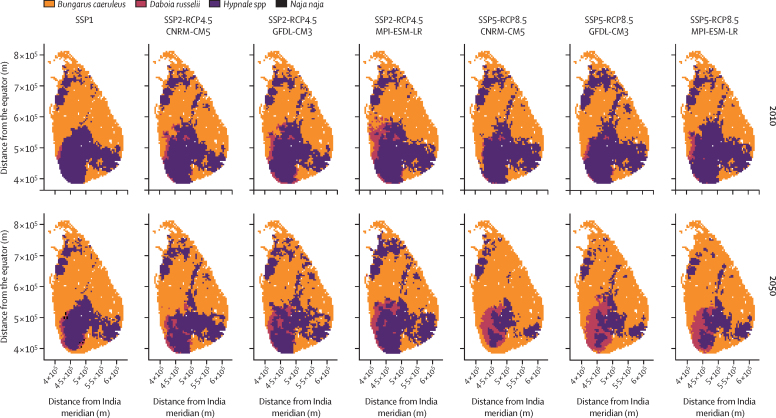
Snake species predicted to be most abundant in each spatial unit in 2010 and 2050 under different SSPs and RCMs Abudance was weighted by the estimated coefficients for each species (*c*_s_ and *b*_s_). Weighted snake abundance outputs used in the snakebite incidence model for all species are shown in the appendix (pp 15–21). Geographical coordinates are from the SLD99 coordinate system for Sri Lanka; the x-axis origin is the central meridian of India. SSP=shared socioeconomic pathway. RCP=representative concentration pathway. RCM=regional circulation model.

The size of the areas with increasing envenoming incidence, and the predicted percentage change in envenoming incidence, were larger in the SSP2-RCP4.5 scenario than in the SSP1 and SSP5-RCP8.5 scenarios (figure 3, figure 4). Most of the increases in incidence occurred in the central highlands (figure 4; elevation model in the appendix [p 14]). In and around the highlands, *Hypnale spp* (present in wet lowlands and highlands^18^) appeared to be replaced by *D russelli* (present in all climatic zones with peak abundances in areas with intermediate precipitation) and *B caeruleus* (predominantly present in dry climates)^18^ by 2050 (figure 5). These changes might be explained mainly by two factors: (1) climate change might increase environmental suitability and therefore potential abundance for *D russelli* and *Hypnale spp* in the highlands in SSP2-RCP4.5, whereas in SSP5-RCP8.5 there would be a decrease in environmental suitability for *Hypnale spp*, despite *Hypnale spp* being widespread and among the most common biting snakes in Sri Lanka; and (2) increased warming, agricultural expansion, and forest degradation would occur in SSP5, which might decrease environmental suitability for snake species (*D russelii*, *Hypnale spp*, *N naja*, and *T trigonocephalus*) in areas other than the highlands more than in SSP2 (figure 3, figure 5, appendix pp 12–14).

Reflecting the national predictions of envenoming incidence, reductions were projected to occur over large areas (figure 3, figure 4). The greatest decrease in envenoming incidence occurred in SSP5-RCP8.5, followed by SSP2-RCP4.5 and SSP1 (figure 3). Changes were also consistent between RCMs, whereby the areas where all three, two, or one RCM showed decreased incidence were also largest in SSP5-RCP8.5, followed by SSP2-RCP4.5. The area with decreased incidence was smallest in SSP1, but we lacked data on possible future climates under SSP1 to find consensus (figure 4).

The predicted changes in envenoming incidence appeared to reflect the summation of the ecological and mechanistic processes underpinning snakebite envenoming, including differences in the way global change might affect individual snake species. For instance, the distribution of the most abundant venomous snakes was predicted to change. *D russelii* was projected to gradually replace *Hypnale spp* in the southwest in all scenarios by 2050. In SSP5-RCP8.5, *B caeruleus* was projected to replace *Hypnale spp* in the north as the most abundant venomous species (figure 5; appendix pp 15–21). The rearrangement of venomous snake species indicates that the nature of envenoming (ie, the severity of impact and type of injuries or disabilities caused) could also change across Sri Lanka.

**Figure 6:**
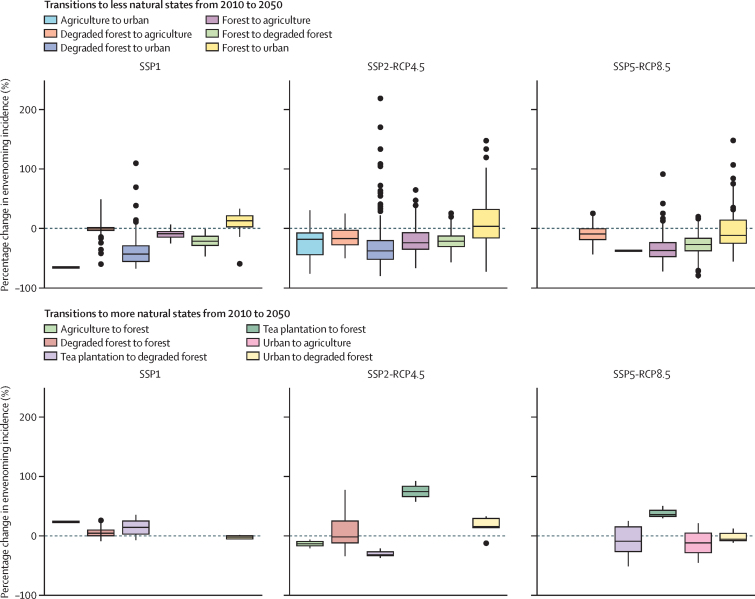
Dispersion of predicted percentage change in envenoming incidence by land cover transitions between 2010 and 2050 in each SSP scenario Predicted percentage change was calculated as: (2050 envenoming incidence – 2010 envenoming incidence) / 2010 envenoming incidence). Boxes show the median and IQR, and whiskers show non-parametric 95% CIs (corresponding to the 2·5th and 97·5th percentiles). Possible transitions that are not shown (eg, urban to forest) did not occur. SSP=shared socioeconomic pathway. RCP=representative concentration pathway.

Land cover transitions to more natural states (eg, degraded forest to forest, and tea plantation to forest) were more likely to increase envenoming incidence than transitions to less natural states (figure 6). Among the scenarios, incidence was in general more likely to increase with transitions to more natural states in SSP1 than SSP2-RCP4.5 and SSP5-RCP8.5; however, transitions were not always directly comparable as the tea plantation to forest transition only occurred in SSP2 and SSP5, and few areas had transitions from agriculture to forest or urban to degraded forest, providing a small amount of data in SSP1. For the corresponding transitions (tea plantation to forest and urban to degraded forest) in SSP2-RCP4.5 and SSP5-RCP8.5, the change in incidence was higher in SSP2-RCP4.5 than SSP5-RCP8.5 (figure 6). Trajectories of land cover change by SSP and RCM are provided in the appendix (p 23).

## Discussion

Contrary to past studies of snakebite risk under climate change, which mostly report patterns of increasing risk,^2^, ^3^ our projections indicated that global change could decrease nationwide snakebite envenoming incidence in Sri Lanka, with the largest decreases predicted under the least sustainable development trajectories (ie, greatest climate and land use change). Nevertheless, incidence was predicted to increase substantially in some local regions (eg, the central highlands) in SSP2-RCP4.5 and SSP5-RCP8.5. The size of the area where incidence would increase was largest in the SSP2-RCP4.5 middle-of-the-road development scenarios (intermediate climate change and high population change). These patterns were consistent regardless of the variability in projected climate, land use, and population trends in the various SSP-RCP4.5 and SSP-RCP8.5 scenarios. The incidence reduction over large areas appeared to be primarily driven by a biodiversity-loss effect, as indicated by the lower potential abundance of four of seven venomous snake species (appendix p 13), whereas localised increases in incidence might have resulted from a combination of low population growth (appendix p 22) and increased climatic suitability for key venomous snake species in the highlands in SSP5-RCP8.5. Our findings suggest that global changes related to climate and human and snake populations will combine to influence human–snake encounters and snakebite incidence in the future.

Accounting for the combined effect of climate, land use, and population changes allows identification of the processes that shape future risk. For example, changes in land cover caused by human activities and climate influence snake habitats and human risk factors.^12^, ^17^, ^18^ Studies have reported decreased envenoming incidence in areas with urban cover due to population growth,^17^, ^19^ whereas we estimated increases in incidence for some land cover transitions to forest cover, likely due to higher suitability of forest cover for snakes (figure 6). In most transitions from more natural to less natural land covers, incidence decreased due to population growth (ie, in SSP1, in which climate is constant). This relationship was evident for transitions to agricultural cover, despite agricultural work being widely recognised as a high-risk occupation^28^ characterised by high human–snake contact rates.^19^ Meanwhile, the abrupt transition from forest cover to urban cover increased envenoming incidence more in the absence of further climate change (SSP1 *vs* SSP2-RCP4.5 *vs* SSP5-RCP8.5, figure 6). These predictions based on process-based models show that understanding how global change components affect each other should be a priority to anticipate changes in snakebite patterns.

Previous assessments, particularly those at large spatial scales, have predicted increased snakebite risk near latitudinal extremes and highlands of tropical regions due to increased climatic suitability for venomous snakes.^3^, ^29^ We found a similar effect related to land elevation, with increased incidence in the central highlands of Sri Lanka, as some snake species will be able to track changes in favourable habitat as temperatures increase by moving up sloped land.^3^, ^30^ However, human population growth, in the Sri Lankan context, has been found to partly eliminate the effect of climate by means of human–snake competition, decreasing snakebite incidence.^17^, ^19^ The alteration of current snake abundance patterns in our predictions is likely to be an example of biodiversity loss.^31^ To estimate the regional and global effects of species loss on snakebite burden and regional incidence profiles, high-resolution incidence data are required from larger areas than Sri Lanka. Predictions for larger areas will help in the development of global schemes for envenoming treatments and preventive measures.^32^ In Sri Lanka, improved measures will be possible with targeted modelling of treatment needs per snake species combined with data on the identity of biting species (published previously^33^).

The forecasts presented in this study should be considered with appropriate caution as they are affected by the complexity and uncertainty of socioecological systems, and by the models used to represent such systems. Key uncertainties in our study include: (1) imperfect detection of snake species in situ, which might affect our estimates of snake abundance and snakebite incidence;^17^, ^18^ (2) incomplete representation of the processes driving snake abundance patterns, such as interactions with other organisms, fine-scale habitat requirements, and other undetected geographical processes such as variability of risk factors between settlements; (3) classification errors or biases in the climate change downscales^24^ (*R*^2^ values in the appendix [p 9]) and land cover predictions;^22^ (4) the propagation of these errors into the snakebite model;^19^ and (5) projection of the snakebite model into future climatic, land cover, and human population conditions. First, with regards to the relevance of our results in relation to snake data deficiencies, the lower abundance of *D russelii* than *Hypnale spp* in the central highlands at baseline in 2010, and the higher abundance for *E carinatus* in areas further inland than near the coast at baseline, are unrealistic^34^, ^35^ (figure 5, appendix pp 15–21). The discrepancies between observations of snake abundance patterns and abundance estimates mean that snakebite envenoming incidence in those regions might differ to our predictions, as *D russelii* and *E carinatus* are a more frequent cause of severe envenoming than *Hypnale spp* if present.^36^ Second, interactions with other organisms are dynamic and irregular across space and time, making environmental effects predominant at medium to large spatial scales.^37^ Third, land cover classification errors, climatic bias errors, and their propagation could impact projections, but the associated uncertainties were alternatively addressed in our scenario analysis via SSPs to capture a range of potential outcomes. Finally, in relation to model transference, the highest risk is climate extrapolation, which could mostly affect *D russelli* in SSP2-RCP4.5, and *Hypnale spp* in SSP2-RCP4.5, as evidenced by negative relationships between distance to the niche centroid and time (appendix p 13). As mentioned, the higher abundance of *E carinatus* inland is dubious*.*^18^, ^34^, ^35^ However, as we generally observed tendencies for climate suitability to worsen for these species, this possibly minimises the risk of extrapolation (appendix p 13).

With further regards to model transference, additional risks are related to the assumption that all parameters will be constant during the next 30 years. Although this might be true for snake climatic and general habitat requirements,^38^ it might not be realistic for socioeconomic risk factors such as barefoot farming and sleeping on the floor. Identifying the parameters that are likely to change over time can help to meet the aims set by WHO's global strategy for prevention and control of snakebite envenoming. For example, decreasing the probability that bites are envenoming is possible with the use of personal protective equipment or mechanising agriculture. In the Sri Lankan context. mechanising rice paddy farming will help decrease *D russelii* bites, and the acquisition of raised beds, mosquito nets, and improved housing will help mitigate risk from *B caeruleus.*^39^ In addition, some of the SSPs might be correlated with mechanisation or prophylaxis which, if measured, will help to fine-tune model parameters and design model-based interventions for the prevailing SSPs. These developments require close epidemiological monitoring, which will further help to identify the health consequences of development pathways and increase data availability for future disease studies.

Despite such limitations, our model provides a novel advance in terms of capturing the key elements driving snakebite envenoming incidence under global change. Our results are consistent with the steady decrease in snakebite-associated mortality observed globally during 1990–2019,^40^ and suggests this trend will continue. The data further provide critical insights, particularly with respect to potential ecological explanations, as to why snakebite incidence is decreasing.

Aside from the incidence of snakebite envenoming, the predicted impacts of global change on snakebite suggested by our study point to other concerning trends, both from an epidemiological perspective and for other disciplines. In ecosystems, snakes are predators, critical for ecosystem function and resilience.^41^ Their conservation and monitoring is thus relevant for environmental and health sustainability. Conservation of venomous snakes might be achieved by creating and protecting the wide diversity of habitats they inhabit.^42^ Although these actions could have positive impacts for ecosystems and societies via the ecosystem benefits and services they provide, we cannot ignore that they might also preserve an ecosystem disservice in the form of snakebite. The same or related mechanisms could similarly apply in the case of other animal-related diseases, including some vector-borne and zoonotic diseases. Therefore, health and environmental agencies should work closely to evaluate and design interventions that can safeguard both human health and the environment, such as unified health and environmental impact assessments, and human–wildlife conflict management strategies. High-income countries such as Australia and the USA, with high venomous snake diversity but low snakebite burden,^43^ highlight how the conflict elsewhere can be managed in the future. Examples of management strategies include increasing the use of personal protective equipment in snake-prone settings, shaping people's knowledge and behaviour when encountering snakes through behavioural campaigns, and ensuring appropriate levels of post-bite treatment access and quality of care. In this way, reducing the impact of snakebite as an ecosystem disservice^39^ while retaining the ecosystem services of snakes (eg, pest regulation in agriculture, and pharmaceutical applications of snake venoms, among other measures) could occur in parallel.

In summary, the interdependence between climate, land use, and social processes affects the dynamics of complex socioecological systems, with consequences for the distribution and burden of human diseases. The health outcomes of rapid global change depend on society's development trajectories, ecological footprint, and human risk factors.^12^ In this study, we showed that global change could result in health benefits in the form of decreasing snakebite envenoming incidence overall, albeit at the cost of localised increases in snakebite risk as well as decreased abundance of some snake species. Such loss and changes within these integrated systems might have other downstream implications for human health, and are the focus of parallel efforts to reverse environmental degradation and protect human health, such as the EU Nature Restoration Law^44^ and Sri Lanka's Land Degradation Neutrality Targets.^45^ Addressing snakebite incidence and biodiversity loss requires joint efforts of public health and nature conservation agencies to implement sustainable policies.

### Contributors

### Data sharing

The snakebite data and code are available via the Zenodo archive (https://doi.org/10.5281/zenodo.7745949). The DynaCLUE land use data and model configuration are also available via the Zenodo archive (https://doi.org/10.5281/zenodo.11122091).

For the **WHO strategy** see https://www.who.int/health-topics/snakebite/who's-global-strategy-for-prevention-and-control-of-snakebite-envenoming#tab=tab_1

## Declaration of interests

GM and JJE received salary from the UK Medical Research Council (grant number MP/P024513/1) in 2018–19. All other authors declare no competing interests.
