# Supplementary material for: Effects of global change on snakebite envenoming incidence up to 2050: a modelling assessment
Source: Lancet Planet Health. 2024 Aug 7;8(8):e533–44. doi: 10.1016/S2542-5196(24)00141-4 (PMC11327114; doi:10.1016/S2542-5196(24)00141-4)
Supplement: Supplementary appendix [file mmc1.pdf]

THE LANCET
Planetary Health

Supplementary appendix

This appendix formed part of the original submission and has been peer reviewed. We post it as supplied by the authors.

Supplement to: Martín G, Erinjery JJ, Ediriweera D, et al. Effects of global change on snakebite envenoming incidence up to 2050: a modelling assessment. *Lancet Planet Health* 2024; **8:** e533–44.

Global change effects on snakebite: an assessment of environmental and health sustainability trade-offs in Sri Lanka

Appendix

Expanded methods

Climate change downscale

We corrected bias using WorldClim v2[1] as *observed* climate and the RCM 970-2006 as *predicted* climate. We extracted WorldClim values from a 15 km radius at the centre of each RCM grid cell, and then calculated the difference between minimum and maximum temperatures ($T_{bias} = T_{observed} - T_{predicted}$) and the relative difference for precipitation ($R_{bias} = R_{observed}/R_{prediced}$)[2] to avoid negative values. Then we averaged the bias factors by month so that we obtained 12 bias correction factors for each month's temperature and rainfall per RCM and RCP.

To estimate absolute climate changes per year, first we corrected bias of the regional circulation model projections of 2006-2050 with the reverse arithmetic, substituting $T_{predicted}$ and $R_{predicted}$ with the RCM values of 2006-2050, so that bias-corrected climate is $T_{corrected} = T_{predicted} + T_{bias}$, and $R_{corrected} = R_{bias} \times R_{predicted}$. Then temperature and rainfall changes per month (ΔT and ΔR) with respect to observed historical climate (monthly averages of WorldClim[1]) are: $\Delta T = T_{corrected} - T_{observed}$, $\Delta R = R_{corrected}/R_{observed}$[2].

Resulting monthly changes were interpolated with thin plate splines from their original resolution of 55 km to the target resolution of 1 km ($\Delta T_{55\ km} \longrightarrow \Delta T_{1\ km}$, $\Delta R_{55\ km} \longrightarrow \Delta R_{1\ km}$)[3], to match WorldClim's 30" dataset [1]. To improve the interpolations over Sri Lanka, we extended the geographical extent by 5° north and west from Sri Lanka including some values over southern India. Once changes were interpolated, we performed the reverse arithmetic used to correct biases, transforming interpolated changes to temperature and rainfall estimates: $T_{predicted,\ 1\ km} = T_{observed,\ 1\ km} + \Delta T_{1\ km}$; $R_{predicted,\ 1km} = R_{observed,\ 1\ km} \times \Delta R_{1\ km}$. Using relative changes for rainfall did not guarantee that all values were positive, as thin plate splines can still generate negative values. Hence we forced all negative values to be equal to the bottom 1st percentile of positive values, and to limit the prediction of extreme values we forced all values larger than the 99th percentile to be equal to that same value for each year, to allow annual variability of extreme rainfall[2].

To validate downscales we calculated the root mean squared error (RMSE), correlation coefficient and linear regression intercept and slope for the predictions between 1970-2006 with the matching climate from the Climate Research Unit v4.03 (CRU)[4]. With the downscaled 1970-2006 RCM predictions we extracted and averaged monthly values from a 15 km radius at the centre of each CRU grid cell from 1 km downscales. Then, we calculated the mentioned statistics. The aim of the described tests was to obtain different measures of the size and type of errors of the predictions: RMSE should be close to zero (0); correlation coefficient positive, close to 1 and significantly different from zero (0); linear regression intercept not significantly different from zero (0) and slope as close as possible to 1 and significantly different from zero (0). Given the lack of available RCP 2.6 in CORDEXSA we used current climate to represent a no-further climate change sustainability pathway (see Figs. 1 and 2).

Land Cover

Base land cover layer

We used Landsat images to produce the base land cover layer of Sri Lanka. We selected the best available surface reflectance level 2 images (Landsat 4-5 TM and Landsat 7ETM+) for the year 2010 with cloud and haze cover <10% (dry season)[5–7] for our classification from https://earthexplorer.usgs.gov/. In the case of Landsat 7ETM+ and regions with low quality images we filled the stripping gaps, finding the missing values in the images closest in time. Once these images were cleaned, we extracted the optical and NDVI bands to classify into discrete classes.

The classification routine used was the Iterative Self-Organizing Data Analysis Technique Algorithm (ISODATA) and visual interpretation of the clusters[8,9]. We ran the ISODATA algorithm with 80 iterations to classify into 40 cluster classes in ENVI version 5.4 and confirmed that all clusters converged within 80 iterations. Then, we refined the classification merging the different cluster classes into the five categories with visual interpretation: *forest*, *degraded forest* (dominated by plantations, fruit orchards and degraded forests), *paddy* (*agriculture*, also included other open field and water-fed crops), *urban* (built up area) and *tea*. To refine classification of the *urban* category we used nightlights data (DMSP-OLS v4.0 and VIIRS v2.0 data; downloaded from NOAA website https://ngdc.noaa.gov/eog/download.html).

Finally, we validated our classification using the ground truth points obtained by visual interpretation of high resolution Google Earth and Google Street view images and expert opinion. We chose 600 random points around Sri Lanka and drew polygons in areas where we could identify homogeneous land cover (images available for year 2010). The overall accuracy of the classification was ~ 95%.

Land cover change simulation modelling

First, we set class transitions rules derived from observed changes between 2010 – 2017 as: *forest* ↔ *degraded forest* ↔ *agriculture* ⟶ *urban*, and depending on the location *forest* ↔ *degraded forest* ↔ *tea* ⟶ *urban*. These transition rules, population growth and the area demanded from each land cover class define the different SSPs: In SSP1 demand of *urban* decreased, and favoured *forest* recovery; SSP2, *agricultural* lands grew but mainly at the expense of *degraded forest*; and in SSP5 *urban* areas and *agriculture* grew at the expense of *forest* and *degraded forest*. To represent the effect of governance on land use change dynamics, we forced natural protected areas to experience zero deforestation under SSP1 and SSP2, and to be open to deforestation under SSP5.

Second, annual rates of class changes were estimated with the observed changes in the 2010-2017 period and assume that these remain constant over time. Third, we selected a set of dynamic (that change with time, Table 2) and static (Table 3) environmental variables that were used to model the probability of presence of each land cover class with a logistic regression (hereafter called location factors). Fourth, we estimated class demands using the total area covered by each land cover class using the Land Use Harmonization (LUH) data[10,11]. For consistence with the base land cover layer, we merged the corresponding LUH classes to represent the above five classes (Table S5). The LUH data represent land cover as proportion of each class in 0.25 × 0.25° pixels in a time

series to year 2100. Therefore the demand corresponds to the sum of %cover × pixel area. As a result this data consists of a table with area values arranged in five columns (land cover classes) and 41 rows (time steps). To allocate the demanded areas for each class, Dyna-CLUE uses the location factors (Tables S2-3) and class transition rules to decide where and how much land of each class will be converted to another.

Finally, we specified spatial restrictions to land cover transitions with a raster layer of distance to the edge of natural protected areas (for SSP1 and 2). Together, land cover demands, location factors and spatial restrictions define the SSP that are represented by land cover predictions. For instance, climatic dynamic factors relate to climate change via the RCP downscales used in projecting the location factors, while human population growth correspond directly to SSPs.

To determine land cover transition rules we analysed the land cover changes between 2010 and 2017 across Sri Lanka. These transition rules take place interactively with the location factors estimated with logistic regressions. These regressions used the presence or absence of each land cover class in 2010 as dependent variable and the environmental variables in Tables S2-3 as explanatory. The presence or absence data for each land cover class were sampled with randomly generated, spatially-independent points. The final logistic model for each land cover class was selected from a model incorporating all the dynamic and static environmental variables and progressively simplifying it until we minimised the Akaike information criterion. The coefficients estimated for each of the final regression models, are those used by Dyna-CLUE as location factors (Table S4). An example of the importance of location factors is that the transition *degraded forest → tea* only occurs in the central highlands (Fig S2), represented by elevation and rainfall variables.

Tree Cover

We allowed a number of tree cover transitions: 5 ↔ 4 ↔ 3 ↔ 2 ↔ 1; 5 →1, 2, 3; 4 → 3, 2, 1; 3 → 2, 1; and reversible 2, 3 → 4; 3, 4 →5. Reversible transitions were only allowed to occur after a period of 20 years, roughly representing the recovery time of vegetation. In scenarios SSP1 and SSP2, tree cover changes were restricted with a raster layer of distance to NPAs. The demand and consequent location factors were calculated with logistic regressions for each tree cover interval comparing tree cover of 2000 with 2010 and with the same dynamic and static location factors as covariables shown in Tables S2-3.

To make tree cover predictions compatible with the original data used as covariates in the snake models, we calculated a correction factor after reclassifying tree cover categories back to proportions, 1 → 0.1, 2 → 0.3, 3 → 0.5, 4 → 0.7, 5→ 0.9. The correction factor (T_{Corr}) was the average difference between the reclassified projected layers and observed tree cover of 2010 in logit scale ($\text{logit}(tc) = \log(tc/1 - tc)$, tc = tree cover): $T_{Corr} = \mathbf{E}[\text{logit}(tc_{proj}) - \text{logit}(tc_{2010})]$, so as to force tree cover predictions to be bounded by 0 and 1. Then the logit-corrected tree cover for year i was $tc'_i = \text{logit}(tc_i) - T_{corr}$, and corrected tree cover is $Tc_i = \exp(tc'_i)/1 + \exp(tc'_i)$.

Tables

Table S1. Correspondence between shared socioeconomic and representative concentration pathways.

SSP	RCP
1 – Sustainability	WorldClim (Current climate†)
2 – Middle of the road	4·5
5 – Fossil fuel development	8·5

†We used current climate instead of RCP 2·6 as CORDEXSA RCMs had not produced such experiments as of September 2019.

Table S2. Dynamic location factors used to influence transition probabilities.

**Variable**	**Source**
Population density from 2010-2050 at 1 km resolution (Totpop)	Jones et al (2016; https://sedac.ciesin.columbia.edu/data/set/popdynamics-pop-projection-ssp-downscaled-1km-2010-2100/data-download) 1 km downscaled human population projections shared socioeconomic pathways (SSP) 1, 2 and 5 to make them compatible with the Gridded Population of the World v4 data (https://sedac.ciesin.columbia.edu/data/set/gpw-v4-basic-demographic-characteristics-rev11/data-download)
Mean Diurnal Range; Mean of monthly (max temp - min temp) (BIO2)	The climatic variables from different GCM's between 2010 and 2050. See section on downscaling of climatic data for more details on how we downscaled the datasets. The variables are named based on WorldClim bioclimatic variables.
Isothermality (BIO3)	
Max Temperature of Warmest Month (BIO5)	
Mean Temperature of Wettest Quarter (BIO8)	
Mean Temperature of driest Quarter (BIO9)	
Mean Temperature of warmest Quarter (BIO10)	
Annual precipitation (BIO12)	
Precipitation Seasonality (Coefficient of Variation); (BIO 15)	
Precipitation of Driest Quarter (BIO17)	
Precipitation of warmest quarter (BIO18)	
Precipitation of coldest Quarter(BIO19)	

Table S3. Static location factors

**Predictive spatial variables**	**Source**
Elevation in meters (Elev)	ASTER global digital elevation model (DEM)at 1 arc sec (downloaded from https://earthdata.nasa.gov) and resampled to 1 km2
SlopeDerivative of ASTER DEM made using spatial analyst extension in ArcGis 10.2	Derivative of ASTER DEM made using spatial analyst extension in ArcGis 10.2
Euclidean distance from road in meters (Distroad)	Derived from shape files provided by Sri Lanka Survey Department (https://www.survey.gov.lk/) at spatial resolution of 1 km^2.
Euclidean distance from tank in meters (Disttank)	
Euclidean distance from cities in meters (Distcities)	
Average income(Avginc)	
Euclidean distance from protected areas in meters (Distpa)	Derived from protected area maps downloaded from world database on protected areas at 1 km^2 spatial resolution (https://www.protectedplanet.net)
Sinhalese population at Gram Niladhari level resampled to 1 Km (Sinhpop)	Originally produced by Sri Lanka Survey Department and resampled to 1 km^2. Obtained from http://riskinfo.lk
Sri Lankan Tamil population at Gram Niladhari level resampled to 1 Km (Sritampop)	
Indian Tamil population at Gram Niladhari level resampled to 1 Km (Indtampop)	

Table S4. Regression models based on dynamic and static factors for Dyna-CLUE models

Model	AUC
$\mathrm{logit}(Forest) = -10.3 + (0.003 \times Elev) + (0.06 \times Slope) + (0.0005 \times Distroad) + (0.00003 \times Disttank) + (-0.0002 \times Distpa) + (-0.0001 \times Sinhpop) + (-0.0009 \times Indtampop) + (-0.002 \times Totpop) + (0.02 \times BIO\,2) + (0.05 \times BIO\,3) + (0.05 \times BIO\,15) + (0.0006 \times BIO\,19)$	0.87
$\mathrm{logit}(degraded) = 0.4 + (-0.002 \times elev) + (-0.05 \times Slope) + (-0.0003 \times Distroad) + (0.00001 \times Distcities) + (-0.00001 \times Avginc) + (0.00003 \times Distpa) + (-0.001 \times Totpop) + (0.03 \times BIO\,2) + (-0.03 \times BIO\,15) + (0.003 \times BIO\,17)$	0.74
$\mathrm{logit}(Paddy) = 38.8 + (-0.01 \times elev) + (-0.0005 \times Distroad) + (-0.0006 \times Distcities) + (0.0008 \times Distpa) + (0.0002 \times sinhpop) + (0.0003 \times Sritampop) + (-0.001 \times Totpop) + (-0.05 \times BIO\,3) + (-0.1 \times BIO\,10) + (-0.0004 \times BIO\,18) + (-0.001 \times BIO\,19)$	0.82
$\mathrm{logit}(Urban) = -14.6 + (-0.3 \times elev) + (-0.00003 \times Disttank) + (0.00006 \times Avginc) + (0.0004 \times Sinhpop) + (0.006 \times Totpop) + (-0.02 \times BIO\,2) + (-0.1 \times BIO\,3) + (0.05 \times BIO\,8)$	0.98
$\mathrm{logit}(Tea) = -0.24 + (-0.001 * Distroad) + (-0.00007 * Avginc) + (0.0001 * Distpa) + (0.0009 * Indtampop) + (0.01 * BIO\,3) + (-0.04 * BIO\,9) + (0.001 * BIO\,12) + (-0.03 * BIO\,15)$	0.98

Table S5. Equivalence between LUH and base line land cover classes

LUH variable	Description	Equivalent land cover class
c3ann	C3 Annual crops	*Agriculture*
c3nfx	C3 Nitrogen-fixing crops	*Agriculture*
c3per	C3 Perennial crops	*Agriculture*
c4ann	C4 Annual crops	*Agriculture*
c4per	C4 Perennial crops	*Agriculture*
pastr	Managed pasture	*Agriculture*
primf	Forested primary land	*Forest*
primn	Non-forested primary land	*Degraded forest*
range	Rangeland	*Agriculture*
secdf	Potentially forested secondary land	*Degraded forest*
secdn	Potentially non-forested secondary land	*Degraded forest*
urban	urban land	*Urban*

Table S6. Average performance statistics of the downscaling method for each regional circulation model compared with observed climate from the Climate Research Unit.

Climate model	Variable	RMSE	r/R^2	Intercept	Slope
CNRM-CM5	Prec	11·81	0·63/0·44	83·1 ($P^\dagger$=0.034)	0·57 (P=0·04)
	Max temp	1·24	0·83/0·68	7·2 (P=0.171)	0·77 ($P = 1·2 \times 10^{-5}$)
	Min temp	1·08	0·91/0·82	7·3 (P = 0.001)	0·69 ($P = 5·6 \times 10^{-8}$)
GFDL-CM3	Prec	122·01	0·62/0·43	86·12 (P = 0.034)	0·68 (P= 0·050)
	Max temp	1·25	0·83/0·68	7·33 (P = 0.170)	0·77 ($P = 6·7 \times 10^{-5}$)
	Min temp	1·07	0·91/0·82	7·4 (P = 0.001)	0·69 ($P = 6·02 \times 10^{-8}$)
MPI-ESM-LR	Prec	115·84	0·64/0·45	80·53 (P = 0.044)	0·62 (P = 0·034)
	Max temp	1·24	0·83/0·68	7·37 (P = 0.171)	0·76 ($P = 4·37 \times 10^{-5}$)
	Min temp	1·07	0·91/0·82	7·39 (P = 0.001)	0·69 ($P = 6·34 \times 10^{-8}$)

†*P*-value estimates obtained with the *t*-statistc.

Table S7. Annual snakebite envenoming changes estimated under each shared socioeconomic pathway and global circulation model. The percentage change shown is accumulated until year 2050.

SSP	Climate model	Estimate	$P^{\dagger}$	% change
SSP1-Historical	WorldClim	-0·0037	5·10 × 10^{-7}	-13·8
SSP2-RCP4.5	CNRM-CM5	-0·0044	4·64 × 10^{-9}	-16·1
SSP2-RCP4.5	GFDL-CM3	-0·0064	1·17 × 10^{-16}	-22·6
SSP2-RCP4.5	MPI-ESM-LR	-0·0032	1·61 × 10^{-5}	-12·0
SSP5-RCP8.5	CNRM-CM5	-0·0058	2.·8 × 10^{-14}	-20·7
SSP5-RCP8.5	GFDL-CM3	-0·0065	2·79 × 10^{-17}	-23·0
SSP5-RCP8.5	MPI-ESM-LR	-0·0064	1·06 × 10^{-16}	-22·6

† *P*-value estimates obtained with the *t*-statistc.

Figures

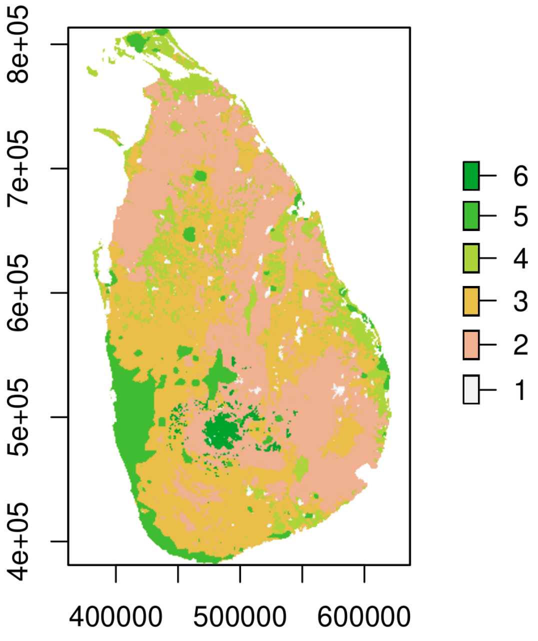

Fig. S1. Base layer of land cover categories upon which land cover predictions were generated. 6 = Tea, 5 = Urban, 4 = Agricultural, 3 = Degraded forest, 2 = Forest, 1 = Water

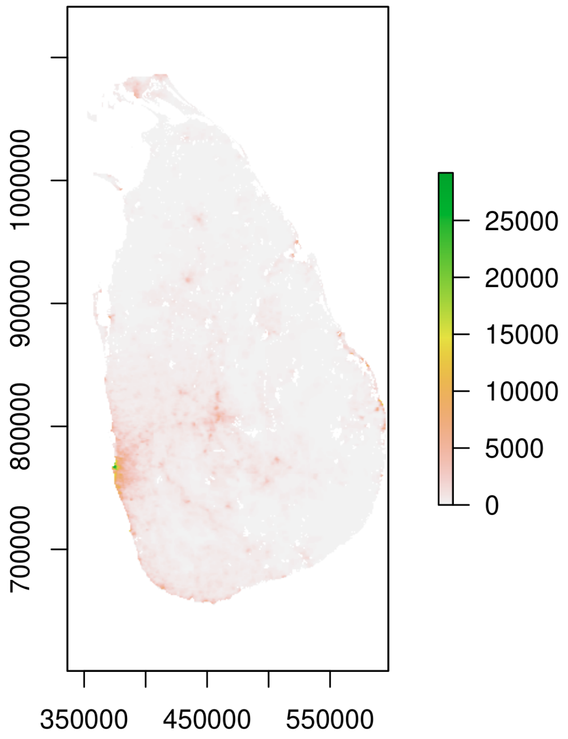

Fig. S2. Human population density of 2010 at 1 km.

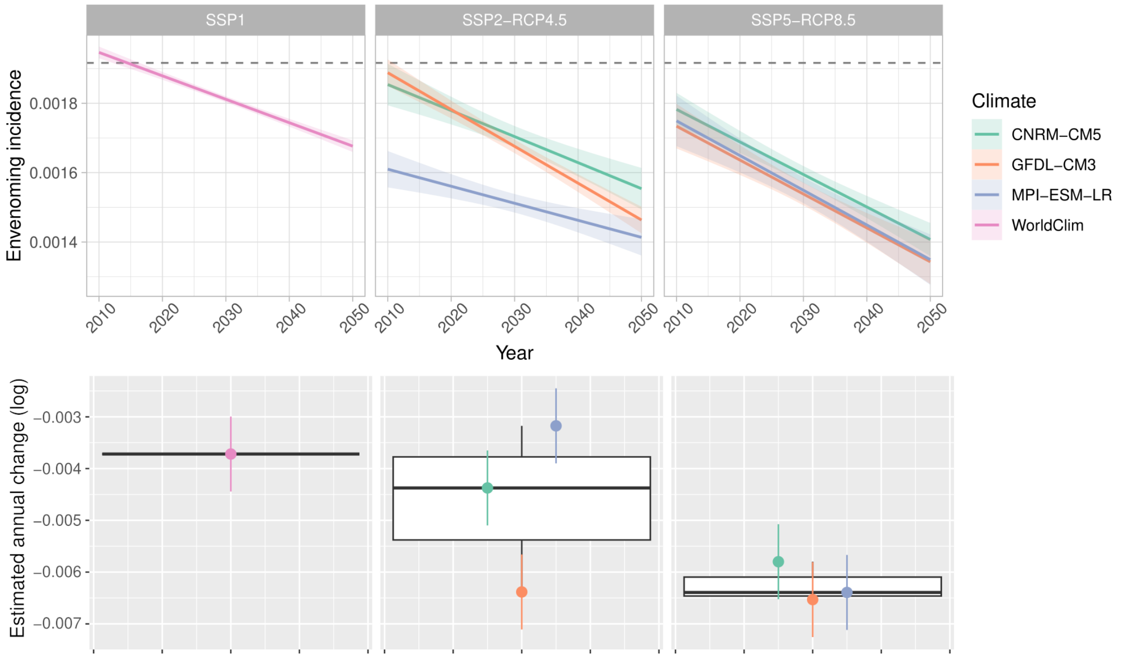

Fig. S3. Predicted envenoming incidence trends between SSPs and among RCMs used (top panels). y-axis scale is national average predicted envenoming incidence. Variability of annual rates of change for each scenario and RCM (bottom panels) where panels indicate the scenario, and colour indicates the climate data or RCM used for projection.

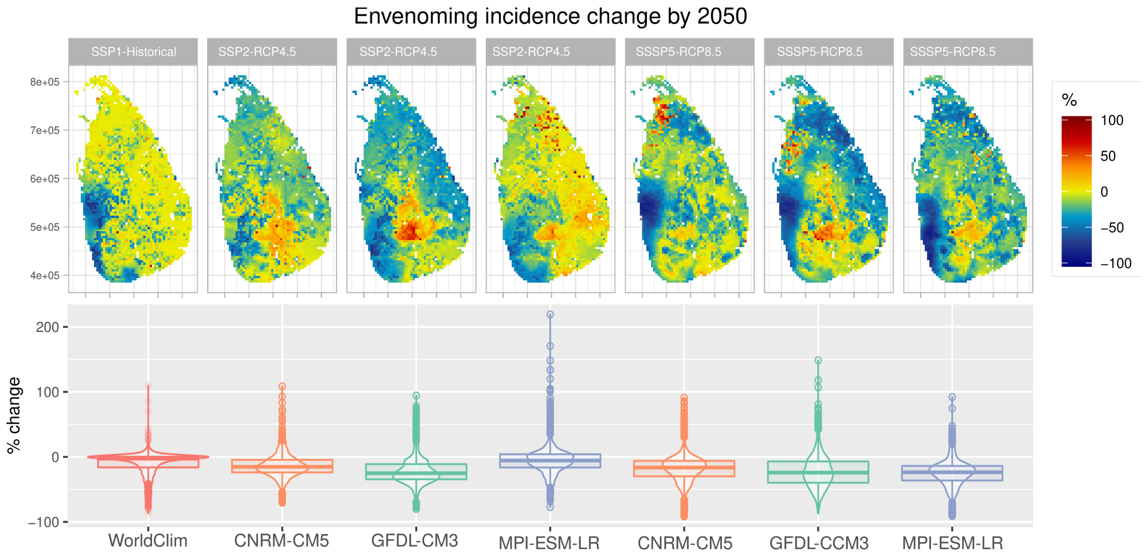

Fig. S4. Percent of change between 2010 and 2050 between scenarios and among climate models (RCMs).

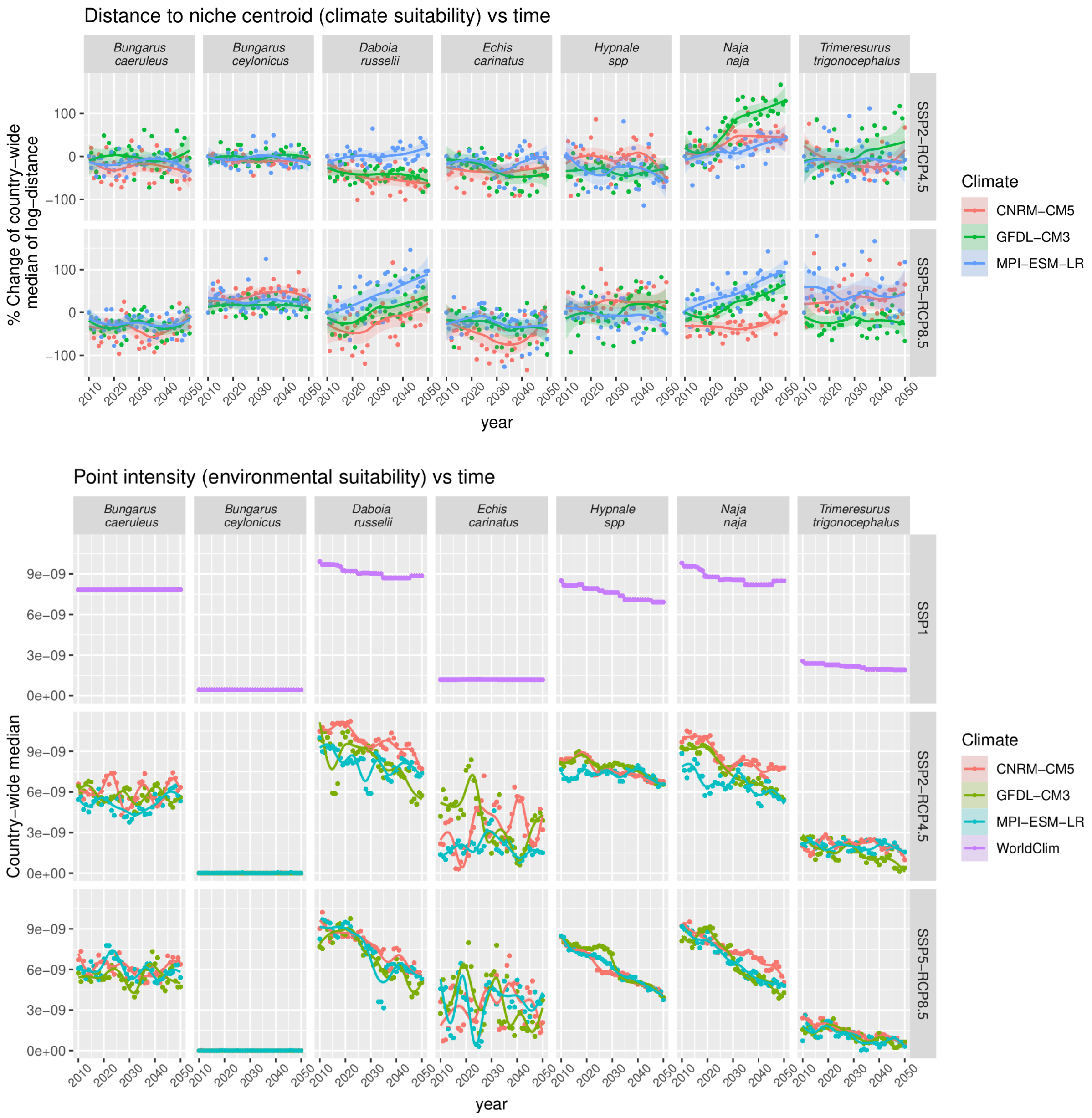

Fig. S5. Percentage of change of the country-wide median of the log distance to each species' niche centroid as a function of time grouped by RCM and SSP-RCP and smoothed (top). The current climate plot has been omitted because it is a straight line. Positive relationships between median distance to the centroid and time implicate that there is lower climatic suitability which translates in lower potential abundance (bottom).

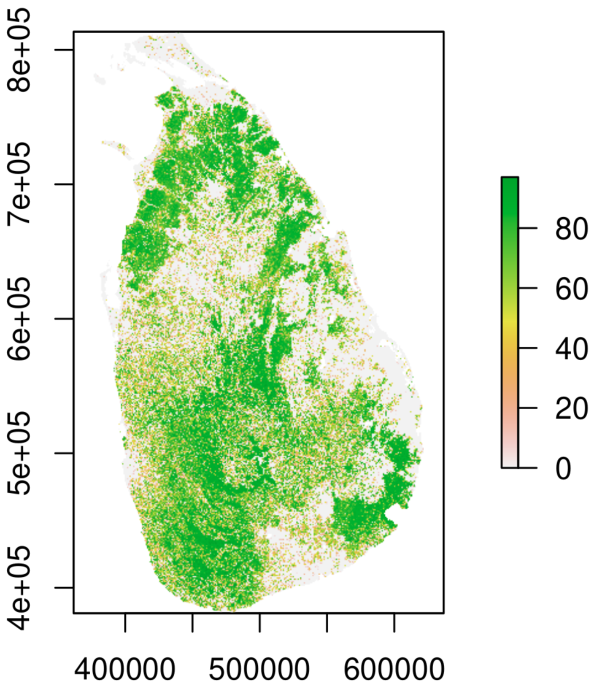

Fig S6. Base tree cover layer used for generating tree cover predictions.

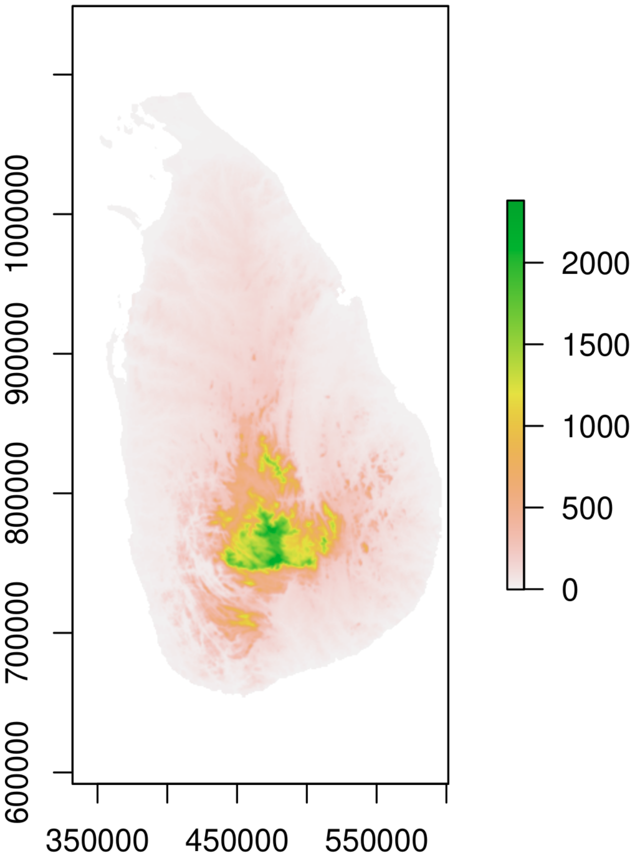

Fig. S7. Digital elevation model of Sri Lanka at 1 km.

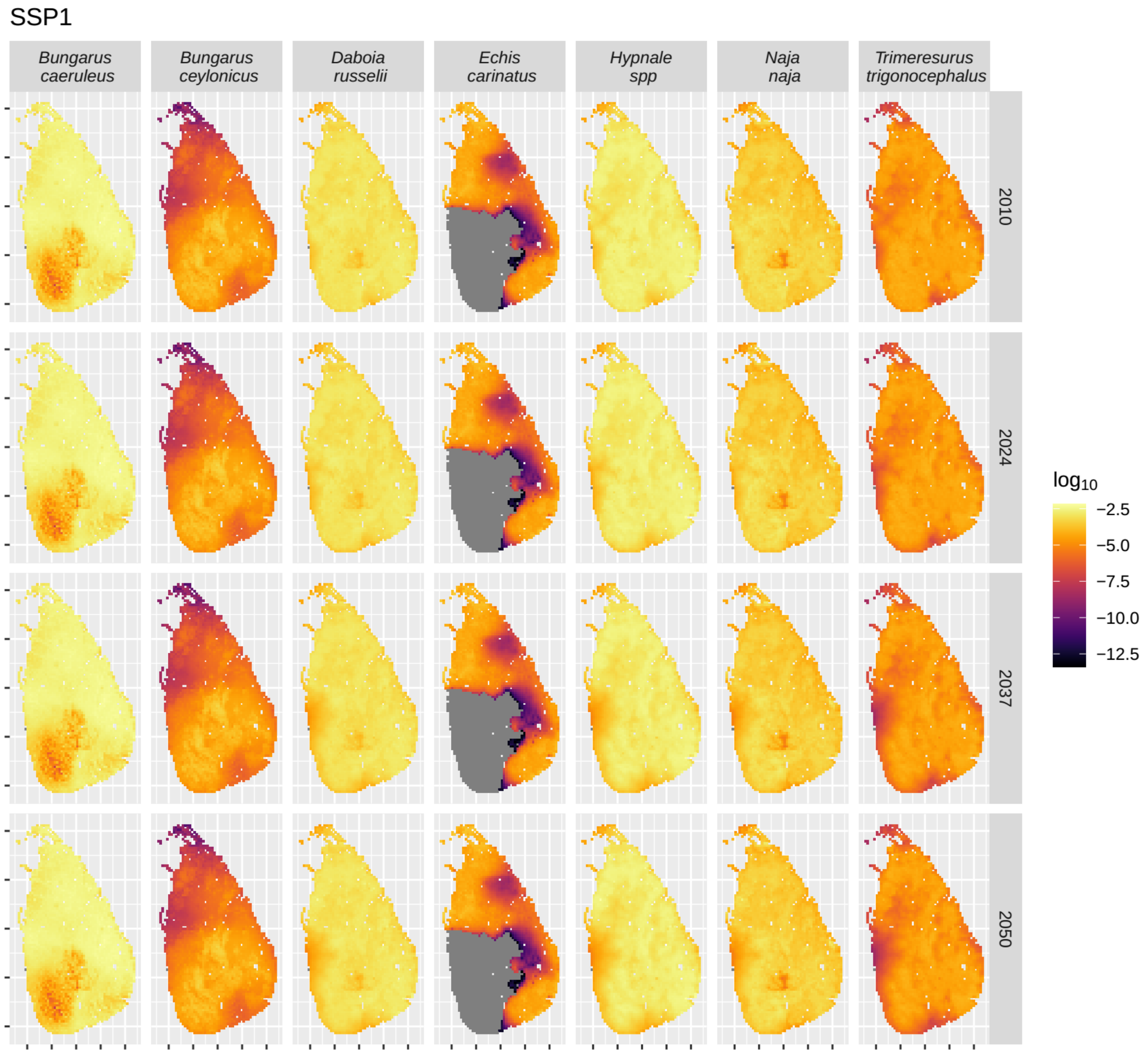

Fig. S8. Snake suitability predictions (potential abundance in $\log_{10}$ scale) corrected for relative abundance for SSP1 in four time steps. Grey colour indicates total absence of suitable conditions.

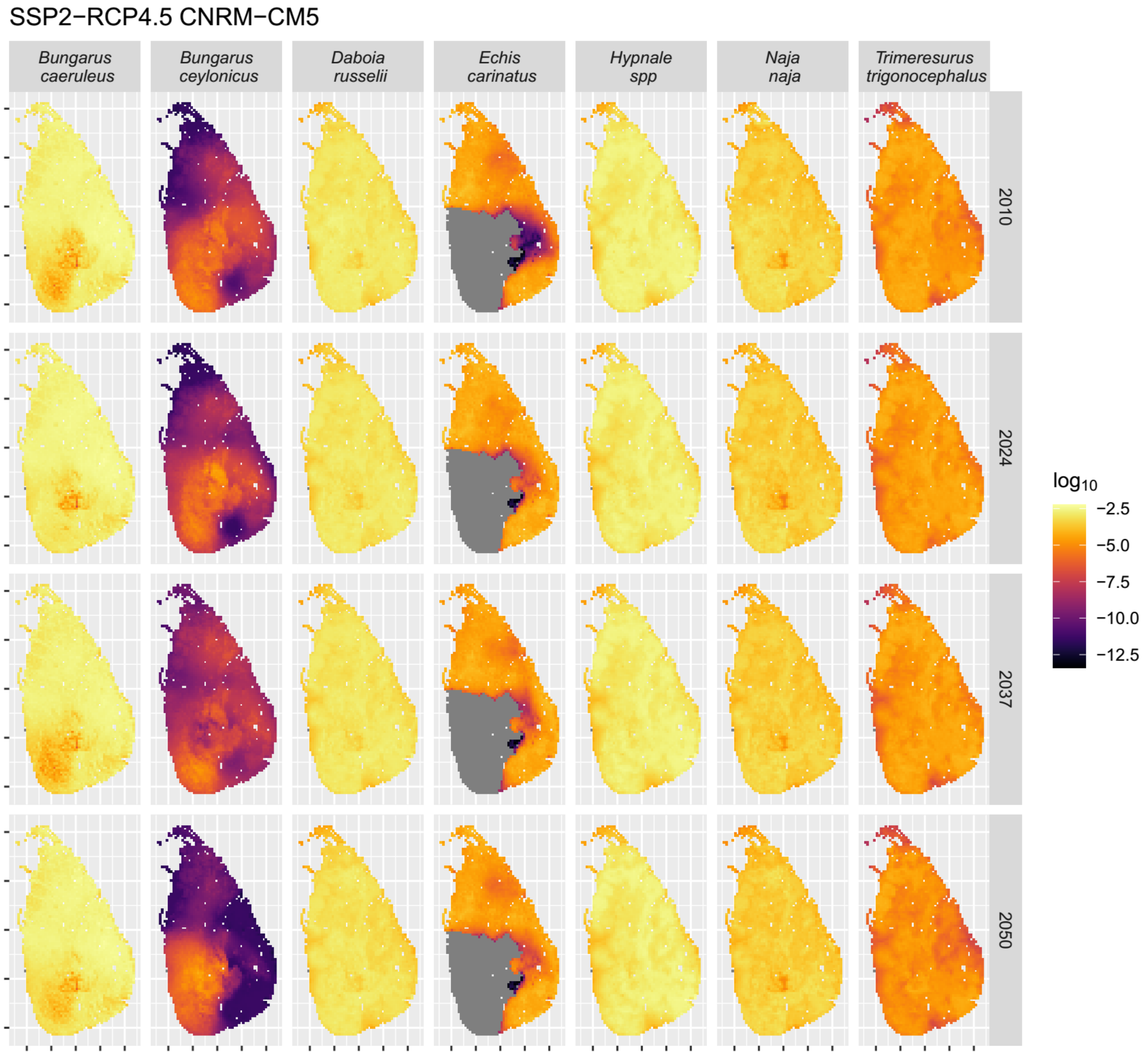

Fig. S9. Snake suitability predictions (potential abundance in $\log_{10}$ scale) corrected for relative abundance for for SSP2 in four time steps (rows) in RCM CNRM-CM5. Grey colour indicates total absence of suitable conditions.

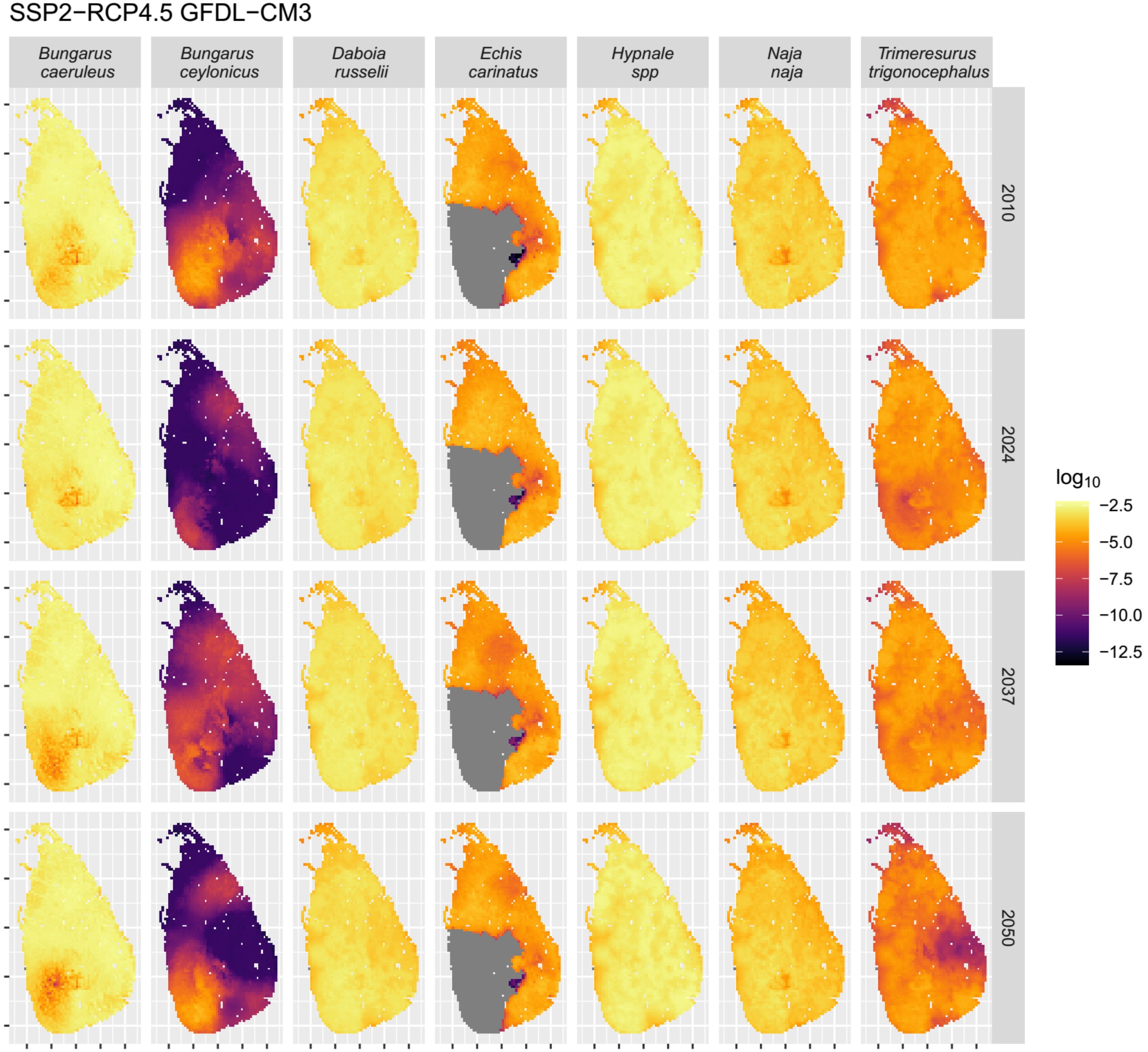

Fig. S10. Snake suitability predictions (potential abundance in $\log_{10}$ scale) corrected for relative abundance for SSP2 in four time steps (rows) in RCM GFDL-CM3. Grey colour indicates total absence of suitable conditions.

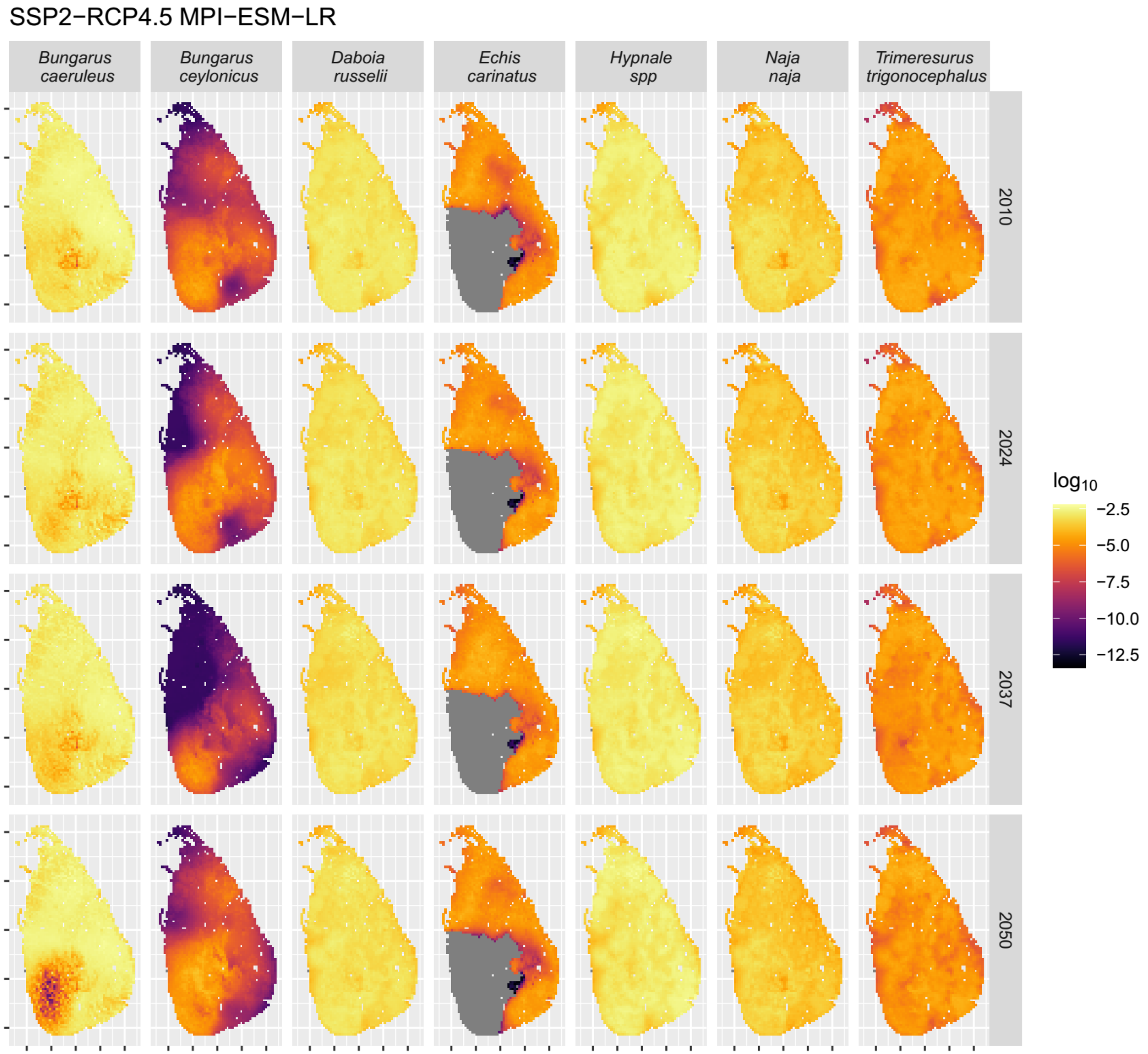

Fig. S11. Snake suitability predictions (potential abundance in $\log_{10}$ scale) corrected for relative abundance for SSP2 in four time steps (rows) in RCM MPI-ESM-LR. Grey colour indicates total absence of suitable conditions.

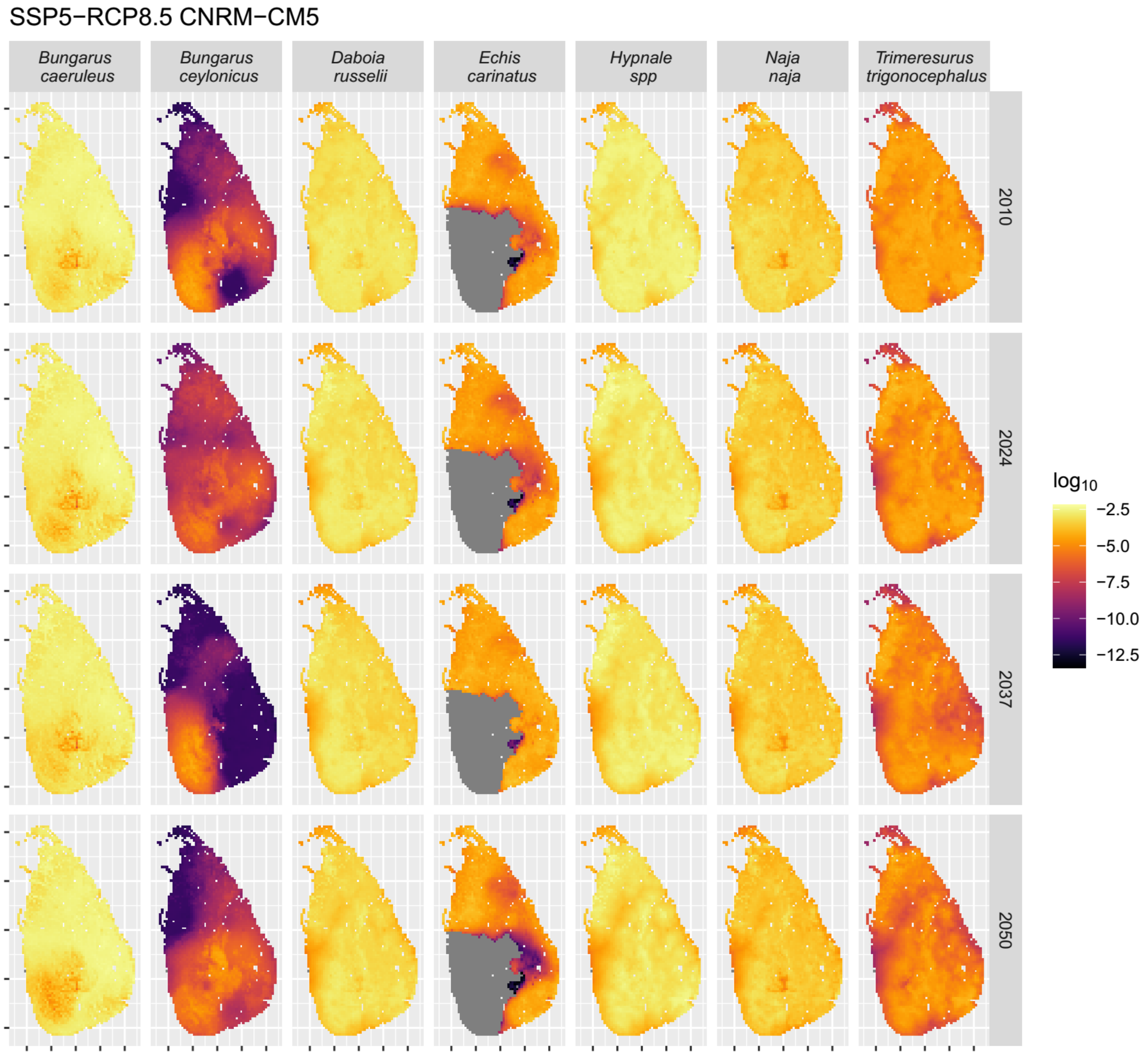

Fig. S12. Snake suitability predictions (potential abundance in $\log_{10}$ scale) corrected for relative abundance for SSP5 in four time steps (rows) in RCM CNRM-CM5. Grey colour indicates total absence of suitable conditions.

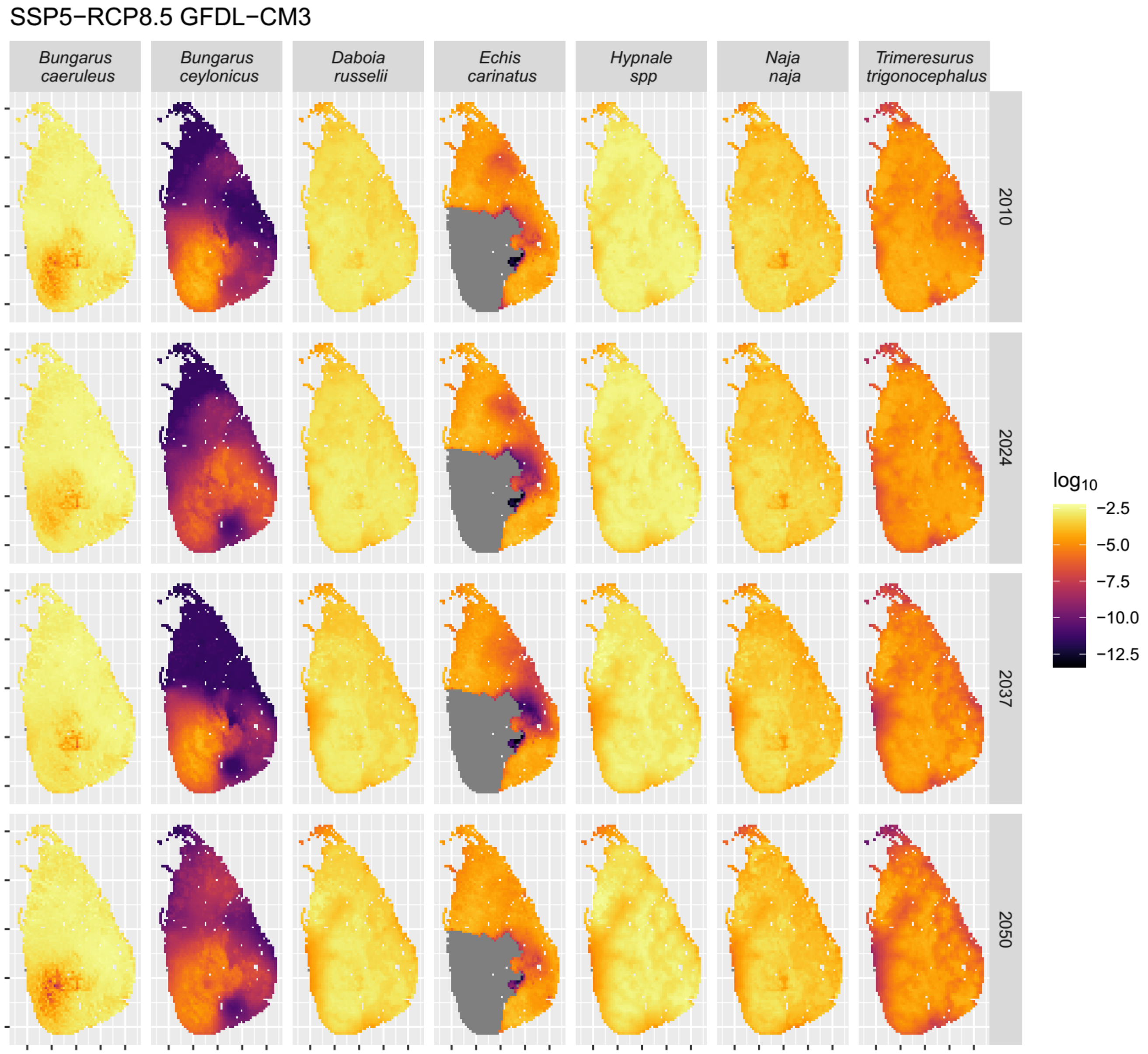

Fig. S13. Snake suitability predictions (potential abundance in $\log_{10}$ scale) corrected for relative abundance for SSP5 in four time steps (rows) in RCM GFDL-CM3. Grey colour indicates total absence of suitable conditions.

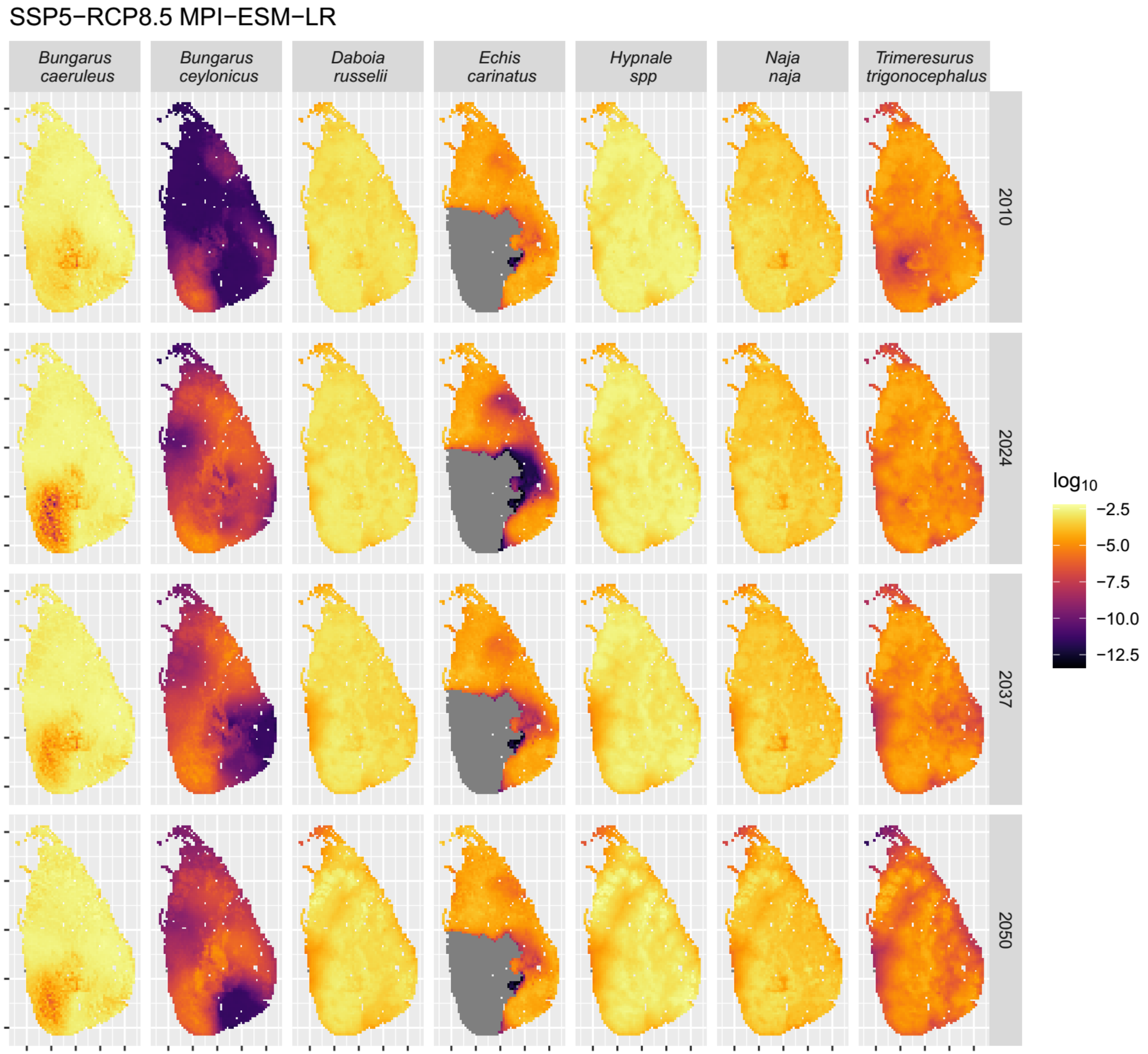

Fig. S14. Snake suitability predictions (potential abundance in $\log_{10}$ scale) corrected for relative abundance for SSP5 in four time steps (rows) in RCM MPI-ESM-LR. Grey colour indicates total absence of suitable conditions.

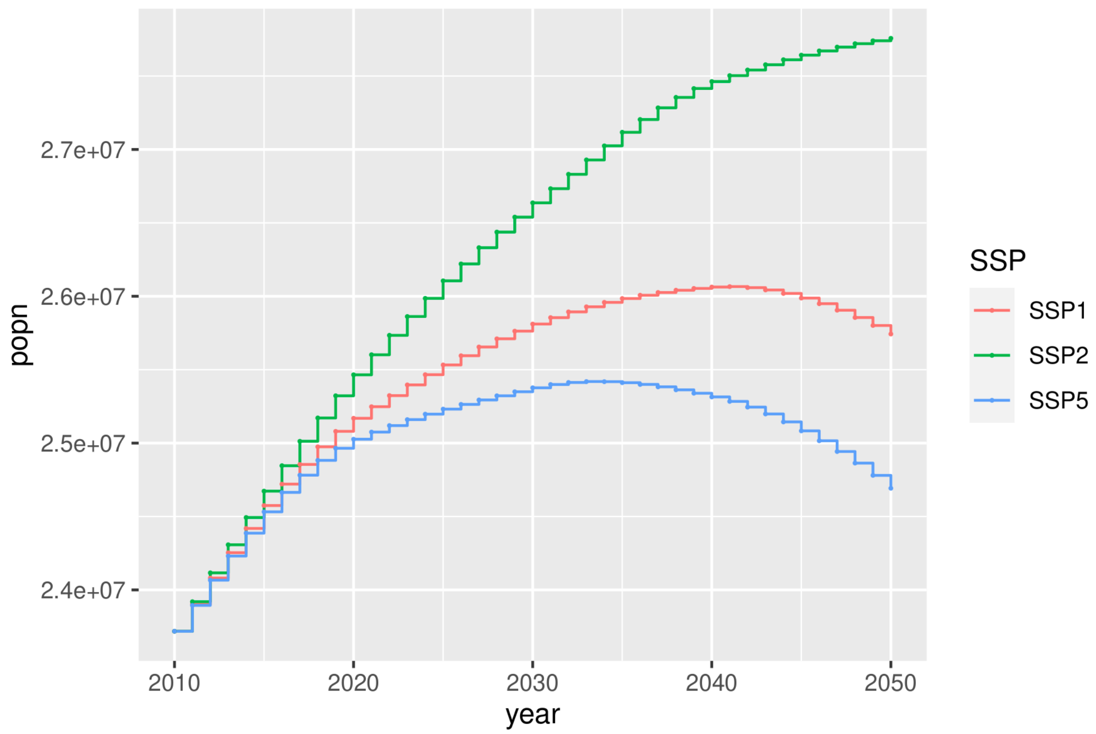

Fig. S15. Population predictions for 2010-2050 in each shared socioeconomic pathway. For reference, SSP1 corresponds to historical climate, SSP2-RCP4.5 and SSP5-RCP8.5.

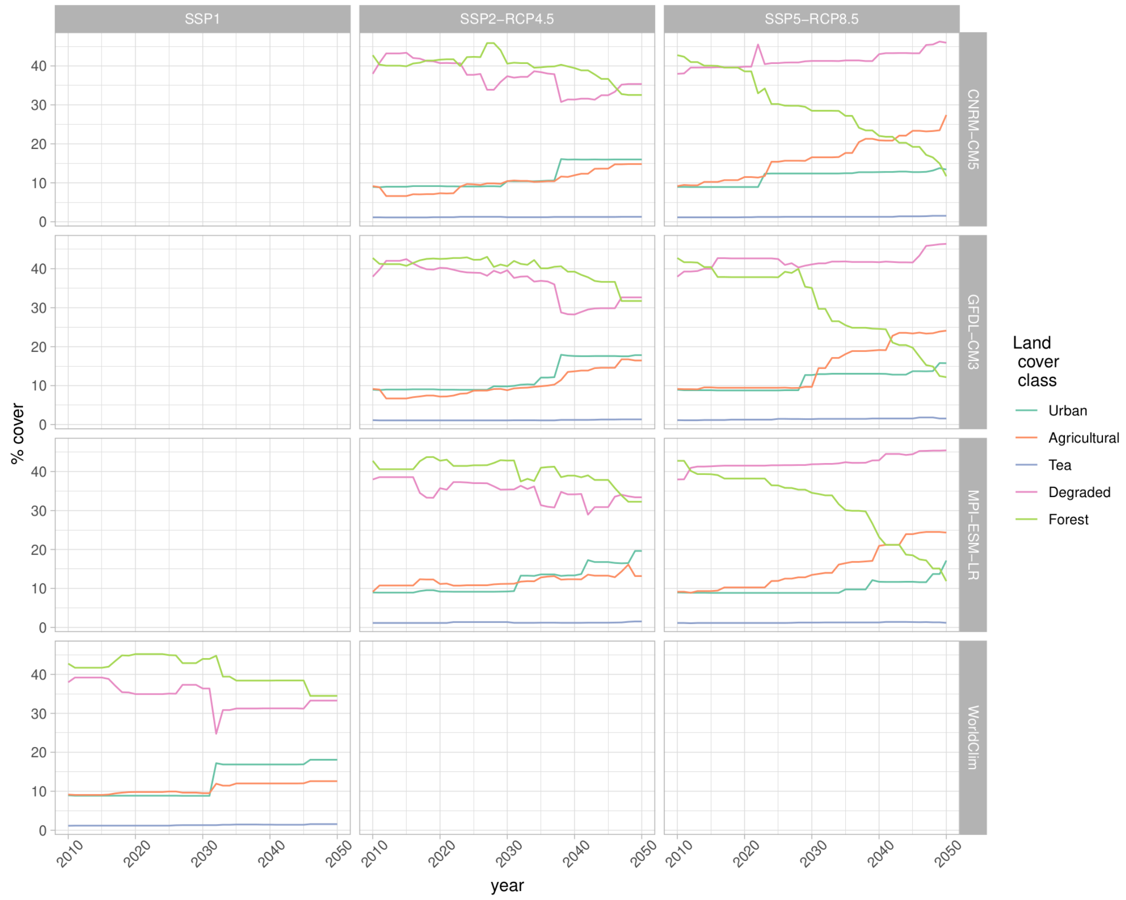

Fig S16. Trajectories of the percentage of land cover in each scenario and with each of the climate models.

References

1 Fick SE, Hijmans RJ. WorldClim 2: new 1-km spatial resolution climate surfaces for global land areas. *Int J Climatol* 2017; **37**: 4302–15.

2 Mosier TM, Hill DF, Sharp KV. 30-Arcsecond monthly climate surfaces with global land coverage: 30-ARCSECOND GLOBAL CLIMATE SURFACES. *Int J Climatol* 2014; **34**: 2175–88.

3 Hijmans RJ, Cameron SE, Parra JL, Jones PG, Jarvis A. Very high resolution interpolated climate surfaces for global land areas. *International Journal of Climatology* 2005; **25**: 1965–78.

4 Harris I, Jones PD, Osborn TJ, Lister DH. Updated high-resolution grids of monthly climatic observations - the CRU TS3.10 Dataset: UPDATED HIGH-RESOLUTION GRIDS OF MONTHLY CLIMATIC OBSERVATIONS. *Int J Climatol* 2014; **34**: 623–42.

5 Gorsevski V, Kasischke E, Dempewolf J, Loboda T, Grossmann F. Analysis of the Impacts of armed conflict on the Eastern Afromontane forest region on the South Sudan — Uganda border using multitemporal Landsat imagery. *Remote Sensing of Environment* 2012; **118**: 10–20.

6 Hayes D, Sader S. Comparison of change-detection techniques for monitoring tropical forest clearing and vegetation regrowth in a time series. *Photogrammetric Engineering and Remote Sensing* 2001. https://www.semanticscholar.org/paper/Comparison-of-change-detection-techniques-for-and-a-Hayes-Sader/5e41444e9c5d03bd7453b68ddeb289d778272ed8 (accessed April 30, 2024).

7 Wulder MA, Skakun RS, Kurz WA, White JC. Estimating time since forest harvest using segmented Landsat ETM+ imagery. *Remote Sensing of Environment* 2004; **93**: 179–87.

8 Camilleri S, De Giglio M, Stecchi F, Pérez-Hurtado A. Land use and land cover change analysis in predominantly man-made coastal wetlands: towards a methodological framework. *Wetlands Ecol Manage* 2017; **25**: 23–43.

9 Stevens K, Campbell L, Urquhart G, Kramer D, Qi J. Examining complexities of forest cover change during armed conflict on Nicaragua's Atlantic Coast. *Biodivers Conserv* 2011; **20**: 2597–613.

10 Chini LP, Hurtt GC, Sahajpal R, *et al.* LUH2-GCB2019: Land-Use Harmonization 2 Update for the Global Carbon Budget, 850-2019. 2021. DOI:10.3334/ORNLDAAC/1851.

11 Hurtt GC, Chini LP, Frolking S, *et al.* Harmonization of land-use scenarios for the period 1500–2100: 600 years of global gridded annual land-use transitions, wood harvest, and resulting secondary lands. *Climatic Change* 2011; **109**: 117.
